# 1,8-Dihydroxy Naphthalene—A New Building Block for the Self-Assembly with Boronic Acids and 4,4′-Bipyridine to Stable Host–Guest Complexes with Aromatic Hydrocarbons

**DOI:** 10.3390/molecules28145394

**Published:** 2023-07-14

**Authors:** Chamila P. Manankandayalage, Daniel K. Unruh, Ryan Perry, Clemens Krempner

**Affiliations:** Department of Chemistry & Biochemistry, Texas Tech University, P.O. Box 41061, Lubbock, TX 79409-1061, USA; chamilapriya1984@gmail.com (C.P.M.); ryan.perry@ttu.edu (R.P.)

**Keywords:** boron, boronic acid ester, Lewis acid–base adduct, self-assembly, boronic acid, dative N→B bonding, host–guest complex, π–π stacking, aromatic hydrocarbons

## Abstract

The new Lewis acid–base adducts of general formula X(nad)B←NC_5_H_4_-C_5_H_4_N→B(nad)X [nad = 1,8-O_2_C_10_H_6,_ X = C_6_H_5_ (**2c**), 3,4,5-F_3_-C_6_H_2_ (**2d**)] were synthesized in high yields via reactions of 1,8-dihydroxy naphthalene [nadH_2_] and 4,4′-bipyridine with the aryl boronic acids C_6_H_5_B(OH)_2_ and 3,4,5-F_3_-C_6_H_2_B(OH)_2_, respectively, and structurally characterized by multi-nuclear NMR spectroscopy and SCXRD. Self-assembled H-shaped Lewis acid–base adduct **2d** proved to be effective in forming thermally stable host–guest complexes, **2d** × solvent, with aromatic hydrocarbon solvents such as benzene, toluene, mesitylene, aniline, and *m*-, *p*-, and *o*-xylene. Crystallographic analysis of these solvent adducts revealed host–guest interactions to primarily occur via π···π contacts between the 4,4′-bipyridyl linker and the aromatic solvents, resulting in the formation of 1:1 and 1:2 host–guest complexes. Thermogravimetric analysis of the isolated complexes **2d** × solvent revealed their high thermal stability with peak temperatures associated with the loss of solvent ranging from 122 to 147 °C. **2d**, when self-assembled in an equimolar mixture of *m*-, *p*-, and *o*-xylene (1:1:1), preferentially binds to *o*-xylene. Collectively, these results demonstrate the ability of 1,8-dihydroxy naphthalene to serve as an effective building block in the selective self-assembly to supramolecular aggregates through dative covalent N→B bonds.

## 1. Introduction

**Scheme 1:**
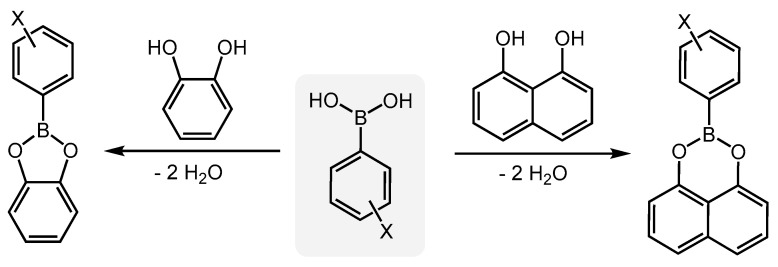
Formation of catechol- and 1,8-dihydroxy naphthalene-based aryl boronic esters.

The last decades have witnessed increased interest in the rational design of supramolecular architectures based on the self-assembly of various suitable building blocks [1,2,3,4,5,6,7,8,9,10,11,12]. Amongst the various strategies, the utilization of intermolecular dative covalent N→B bond interactions in the design and self-assembly of well-defined supramolecular structures has become a promising synthetic approach with potential in host–guest chemistry, gas storage, and crystal engineering [13,14,15,16]. Examples of this recent development are trigonal planar catechol-based aryl boronic esters [17]. These strongly Lewis acidic building blocks, in combination with N-containing strongly Lewis basic ligands, have been demonstrated to facilitate self-assembly to give—through coordinate covalent N→B bonds—boronic ester-based supramolecular structures, such as coordination polymers, cage- and nanostructures, and gels [18,19,20,21,22,23,24,25,26,27]. Recently, the groups of Hőpfl and Morales-Rojas [28] have shown that reactions of catechol-based aryl boronic esters with bipyridine linkers in the presence of various aromatic hydrocarbon solvents readily form self-assembled Lewis-type N→B adducts with aromatic hydrocarbons incorporated. It was hypothesized that upon N→B coordination, the aromatic bipyridine linker becomes increasingly electron-deficient, enabling the recognition and selective isolation of host–guest complexes with aromatic hydrocarbons [29]. However, catecholate-based boronic esters, despite their high Lewis acidity, readily hydrolyze, particularly when used in “wet” solvents. Our group has recently shown that 1,8-dihydroxy naphthalene-based aryl boronic esters are as strongly Lewis acidic as their catechol-based counterparts but significantly more hydrolytically stable (Scheme 1) [30]. Based on our recent findings, we envisioned that 1,8-dihydroxy naphthalene should be an effective building block in the self-assembly with aryl boronic acids and a suitable bipyridine linker to form thermally stable host–guest complexes with aromatic hydrocarbons.

## 2. Results and Discussion

**Scheme 2:**
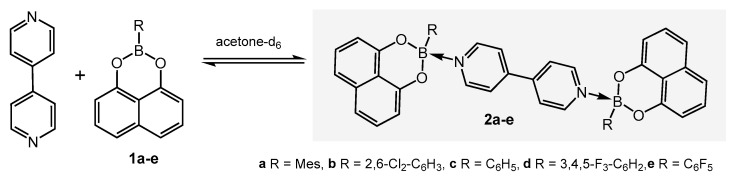
Equilibrium between 4,4′-bipyridine/**1a**–**e** and the Lewis acid–base adducts **2a**–**e** in dilute acetone-d_6_ at room temperature.

**Figure 1:**
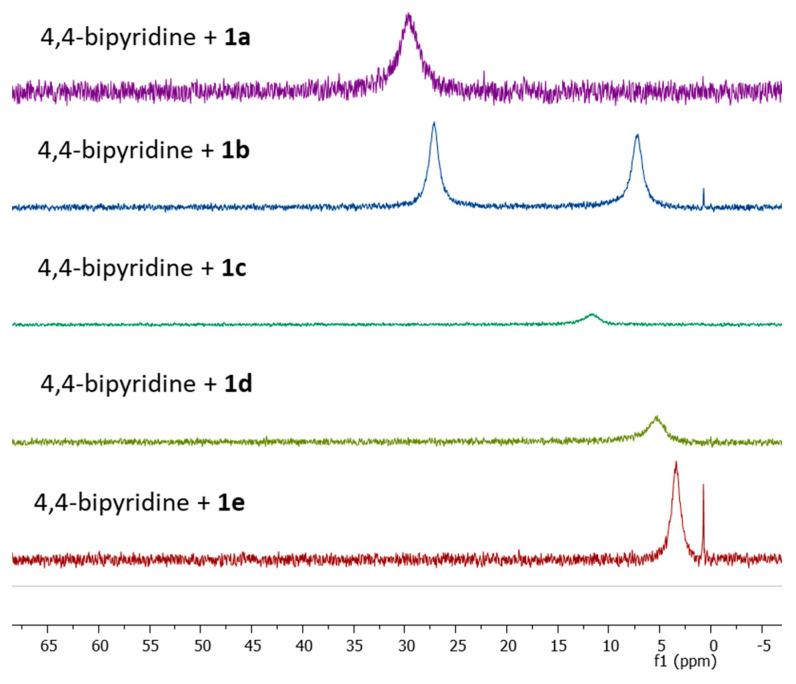
^11^B NMR spectroscopic study of the equilibrium reaction of the esters **1a**–**e** with 4,4′-bipyridine in acetone-D_6_ at room temperature.

At the outset, we investigated the ability of our recently prepared boronic esters of general formula R-Bnad (**1a**–**e**), where nad = 1,8-naphthtalenediolate (**a**: R = mesityl; **b**: R = 2,6-dichlorophenyl; **c**: R = phenyl; **d**: R = 3,4,5-trifluorophenyl; **e**: R = pentafluorophenyl) to form stable Lewis acid–base adducts with 4,4′-bipyridine (Scheme 2). The reactions were performed in acetone-D_6_ as the solvent and monitored by ^11^B and ^1^H NMR spectroscopy. However, the ^1^H NMR spectra of all five reactions exclusively gave one set of signals, even when an excess of 4,4′-bipyridine or one of the respective boronic esters was used, indicating rapid equilibrium to occur at room temperature. The ^11^B NMR spectra, on the other hand, showed very different spectral features (Figure 1). Thus, a 1:2 mixture of 4,4′-bipyridine and the bulkiest and least Lewis acidic ester **1a** (R = mesityl) showed a signal at around 29 ppm, which is close to the ^11^B NMR chemical shift of **1a** (δ = 27 ppm in CDCl_3_) [30], showing that the equilibrium strongly favors the individual acid and base components over adduct **2a**. For the system 4,4′-bipyridine/**1b** (1:2), two distinct signals at ca. 27 ppm and 7 ppm were observed; the former can be assigned to ester **1b**, while the latter is due to the desired Lewis acid–base adduct **2b**. In contrast, mixtures of 4,4′-bipyridine with **1c**, **1d**, and **1e**, respectively, showed up-field shifted signals at ca. 12 ppm (**1c**), 6 ppm (**1d**), and 4 ppm (**1e**), indicating the formation of the respective adducts **2c**, **2d**, and **2e**.

**Figure 2:**
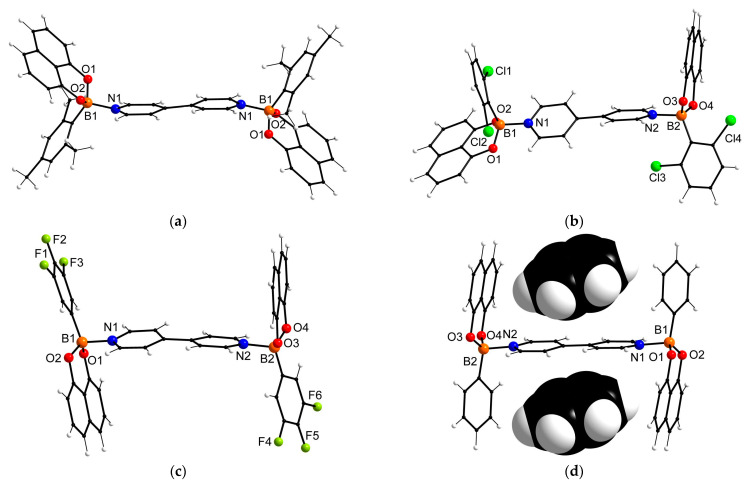
Solid-state structures of the 4,4′-bipyridine adducts **2a**, **2b**, **2d** (co-crystallizing acetone molecules omitted for clarity), and **2c** × benzene. Color codes: red = oxygen, orange = boron, green = fluorine, white = hydrogen, black = carbon, blue = nitrogen. Selected bond lengths [Å] and angles [°]: (**a**) **2a**, O1 B1 1.476(2), O2 B1 1.462(2), N1 B1 1.627(2), O2 B1 O1 110.9(1), O2 B1 N1 104.7(1), O1 B1 N1 100.3(1); (**b**) **2b**, O1 B1 1.448(2), O2 B1 1.449(2), O3 B2 1.453(2), O4 B2 1.442(2), N1 B1 1.611(2), N2 B2 1.650(2), O1 B1 O2 112.1(1), O1 B1 N1 104.9(1), O2 B1 N1 105.4(1), O4 B2 N2 106.2(1), O3 B2 N2 102.5(1), O4 B2 O3 116.2(1); (**c**) **2d**, B1 O2 1.449(2), B1 O1 1.453(2), B1 N1 1.675(2), B2 O4 1.447(2), B2 O3 1.452(2), B2 N2 1.669(2), O2 B1 N1 105.9(1), O1 B1 N1 104.5(1), O2 B1 O1 115.0(1), O4 B2 N2 104.4(1), O3 B2 N2 105.9(1), O4 B2 O3 115.2(1); (**d**) **2c** × benzene, O1 B1 1.455(2), O2 B1 1.456(2), O3 B2 1.458(2), O4 B2 1.453(2), N1 B1 1.673(2), N2 B2 1.670(2), O1 B1 O2 114.1(1), O1 B1 N1 104.8(1), O2 B1 N1 104.4(1), O4 B2 O3 114.1(1), O4 B2 N2 104.9(1), O3 B2 N2 104.5(1).

To confirm our structural assignments, efforts were undertaken to grow single crystals suitable for X-ray analysis from concentrated acetone solutions of 4,4′-bipyridine and **2a**–**e**. The results for **2a**, **2b**, and **2d** are shown in Figure 2 and reveal that two boronic ester units bind to a 4,4′-bipyridine moiety linked via dative N→B bonds. Compounds **2a** and **2b** can structurally be described as double-tweezers. **2a** exhibits an almost perfect double-tweezer-shaped structure with nearly planar bipyridine units, while **2b** is markedly twisted with a twisting angle of the bipyridine moiety of about 35°. Adduct **2d**, on the other hand, is composed of a more H-shaped structure with a nearly planar bipyridine unit (twisting angle ~10°). The bond parameters for all three adducts are similar, with B-N and B-O distances ranging from 1.61 to 1.67 Å and 1.45–1.47 Å, respectively. The central boron atoms in **2a**, **2b**, and **2d** have slightly distorted tetrahedral coordination environments, with O-B-O and N-B-O angles ranging from 111–116° to 102–105°. Unfortunately, it was not possible to obtain suitable single crystals of **2c** and **2e** from acetone as the solvent. Not only was **2e** highly soluble in acetone, but it also slowly degraded to a second product exhibiting a relatively sharp signal in the ^11^B NMR with a chemical shift of ca. 0 ppm (see also Figure 1). This new product crystallized from acetone after a week and was identified by NMR spectroscopy and SCXRD as a spirocyclic 4,4′-bipyridinium borate (see supporting information). However, switching the solvent from acetone to benzene resulted in the formation of single crystalline material, which by X-ray analysis was identified as the phenyl derivative **2c** × benzene (Figure 2). Similar to **2d**, adduct **2c** × benzene has an H-shaped structure with a nearly planar bipyridine unit (twisting angle ~ 5°) and average B-O and B-N distances of 1.45 and 1.67 Å, respectively. Notably, **2c** × benzene co-crystallizes with two molecules of benzene, both being entrapped via π–π stacking with the 4,4′-bipyridine moiety and C-H^…^π contacts with both the phenyl and the naphthalene substituents.

**Scheme 3:**
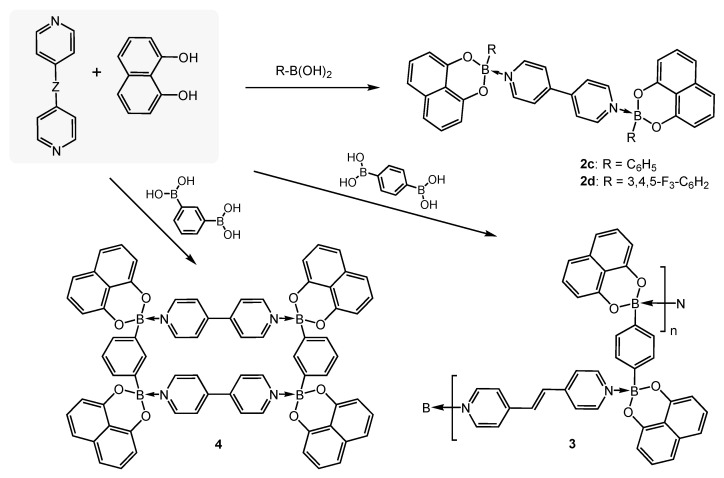
One-pot synthesis of **2c**, **2d**, **3**, and **4**.

It was also possible to synthesize and isolate adducts **2c** and **2d** via a one-pot procedure in excellent yields starting from the respective boronic acids, without the need of preparing the respective esters **1c** and **1d** (Scheme 3). For example, when two equivalents of 1,8-dihydroxy naphthalene and one equivalent of 4,4′-bipyridine were reacted with two equivalents of the respective aryl boronic acid in acetone as a solvent, **2c** and **2d** were isolated in yields of 80% and 90%, respectively, as bright orange crystalline materials. Both compounds were fully characterized by ^1^H, ^13^C, ^19^F, and ^11^B NMR spectroscopy as well as by combustion analysis.

**Figure 3:**
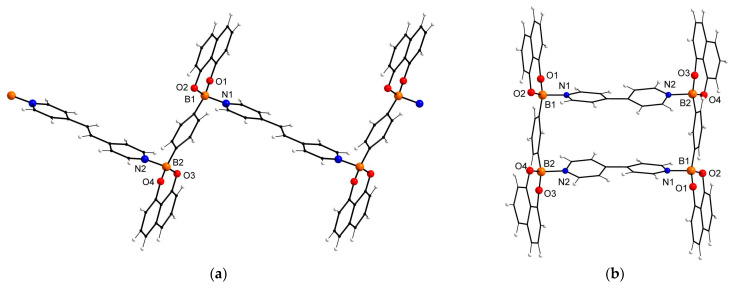
Solid-state structures of **3** (**a**) (disordered DMSO molecules omitted for clarity) and **4** (**b**) (disordered acetone molecules omitted for clarity). Color codes: red = oxygen, orange = boron, white = hydrogen, black = carbon, blue = nitrogen. Selected bond lengths in [Å] and angles [°]: **4**, O1 B1 1.454(2), O2 B1 1.458(2), O3 B2 1.451(2), O4 B2 1.457(2), N1 B1 1.672(2), N2 B2 1.678(2), O1 B1 O2 114.9(1), O1 B1 N1 105.6(1), O2 B1 N1 105.3(1), O3 B2 O4 114.9(1), O3 B2 N2 105.1(1), O4 B2 N2 105.9(1).

Encouraged by these results, the self-assembly with diboronic acids was investigated. Thus, upon reacting 1,8-dihydroxy naphthalene with 4,4′-bipyridine and 1,4-phenylene diboronic acid in a 2:1:1 ratio in acetone as the solvent, an orange crystalline material formed. However, once precipitated, the isolated material proved to be insoluble in common organic solvents, rendering its characterization by NMR spectroscopy impossible. Attempts to grow single crystals suitable for X-ray analysis failed as well. On the other hand, replacing 4,4′-bipyridine with trans-1,2-di(4-pyridyl)ethylene gave— upon reaction with 1,8-dihydroxy naphthalene and 1,4-phenylene diboronic acid in acetone—an orange microcrystalline precipitate, from which single crystals suitable for X-ray analysis could be obtained. The results revealed the compound to be polymeric Lewis acid–base adduct **3**. Polymer **3** is composed of diboronic ester units coordinated with the 4,4′-bipyridine moieties in a zig-zag fashion (Scheme 3, Figure 3a). Due to poor diffraction and multiple twinning of the single crystals measured, only the connectivity of the polymer could be confirmed.

In contrast, the reaction of 1,3-phenylene diboronic acid under otherwise identical conditions did not lead to the formation of a polymeric structure; instead, the rectangular dimer **4** (yield 94%) was formed as confirmed by single-crystal X-ray analysis (Scheme 3, Figure 3b). Both bipyridine units in **4** are markedly twisted, with twisting angles of about 40°. The average B-N and B-O distances are in the expected range of 1.675 and 1.455 Å, respectively.

**Scheme 4:**
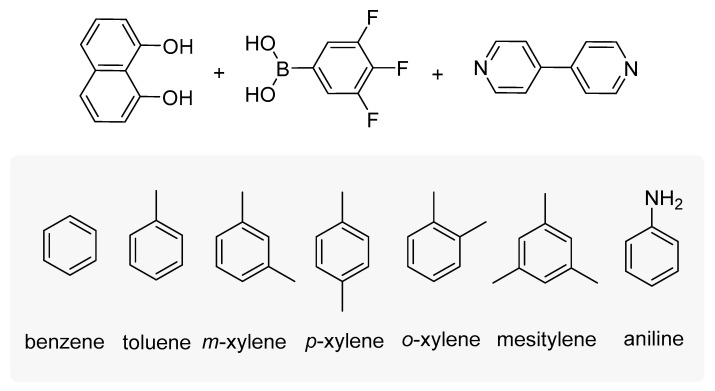
Host–guest chemistry of **2c** with various aromatic hydrocarbon solvents.

Encouraged by the ability of **2c** × benzene to form a stable host–guest complex with two molecules of benzene, a detailed study of the inclusion properties of H-shaped **2d** was undertaken (Scheme 4). **2d** was selected because of its enhanced hydrolytic stability in solution and the ability of the fluorine substituents to significantly decrease the electron density at boron, thereby increasing the stability of the Lewis acid–base adducts to be formed. Suspensions of 1,8-dihydroxy naphthalene, 4,4′-bipyridine, and 3,4,5-trifluorophenyl boronic acid in the respective aromatic hydrocarbon solvent (i.e., benzene, toluene, *o*-xylene, *m*-xylene, *p*-xylene, mesitylene, aniline) were heated until clear solutions were obtained. Upon slowly cooling to room temperature, crystalline solids were obtained in isolated yields ranging from 34–81%. In addition to being characterized by ^1^H NMR spectroscopy and combustion analysis, all compounds were analyzed by X-ray crystallography.

**Figure 4:**
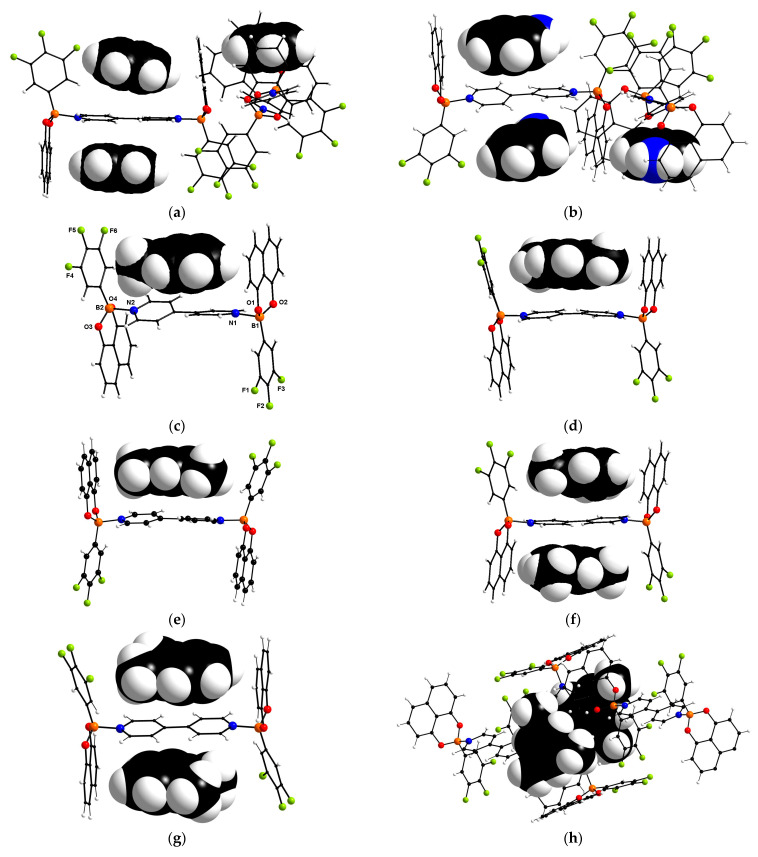
Solid-state structures of host–guest complexes of **2c** with various aromatic solvents (color codes: red = oxygen, orange = boron, white = hydrogen, black = carbon, blue = nitrogen, green = fluorine): (**a**) **2d** × benzene; (**b**) **2d** × aniline, (**c**) **2d** × toluene, (**d**) **2d** × *p*-xylene; (**e**) **2d** × *m*-xylene; (**f**) **2d** × *m*-xylene (unbound *m*-xylene omitted for clarity); (**g**) **2d** × *o*-xylene; (**h**) **2d** × mesitylene (unbound mesitylene omitted for clarity).

The results of the X-ray analysis are shown in Figure 4 and reveal that **2d** is capable of forming various stable host–guest complexes with all aromatic hydrocarbon solvents used in this study via π–π stacking between the 4,4′-bipyridyl unit of **2d** and the respective aromatic solvent. Thus, in benzene as a solvent, **2d** × benzene is formed (Figure 4a), which is composed of three benzene molecules per two molecules of **2d**. Identical results were obtained with aniline as the solvent, generating host–guest complex **2d** × aniline (Figure 4b) with a **2d**/aniline ratio of 2:3. In contrast, carrying out the self-assembly reactions in toluene and *p*-xylene gave the host–guest complexes **2d** × toluene and **2d** × *p*-xylene (Figure 4c,d), respectively, which consist of one aromatic guest per one molecule of **2d** (1:1 complex). Interestingly, crystallization experiments in meta-xylene gave two crystallographically distinct single crystals of **2d** × *m*-xylene; the results for one revealed a classical 1:1 host–guest complex (Figure 4e), while X-ray analysis of the second one revealed a 1:2 complex (Figure 4f) with two additional unbound *m*-xylene molecules per three equivalents of the 1:2 complex. On the other hand, X-ray analysis of crystalline **2d** × *o*-xylene obtained from *o*-xylene as a solvent confirmed a 1:2 complex (Figure 4g). The X-ray analysis for **2d** × mesitylene (Figure 4h) also confirms a 1:2 host–guest complex but with two molecules of **2d** and two molecules of unbound mesitylene in the unit cell.

Table 1 compares the host–guest ratios of **2d** × solvent determined from the X-ray analysis of single crystals with those obtained from the ^1^H NMR data of the bulk materials; the latter were dried in air for circa 24 h. It was found that crystals of **2d** × *o*-xylene and **2d** × *m*-xylene, upon drying in air, lose guest molecules to form host–guest complexes with a formal 1:1 host–guest ratio. While in **2d** × aniline and **2d** × benzene, the 2:3 host–guest ratio remains unchanged, and **2d** × mesitylene contains significantly more mesitylene than what was found via X-ray analysis of single crystalline material. This seems to imply that **2d** × mesitylene may crystallize in various forms with different host–guest ratios.

**Figure 5:**
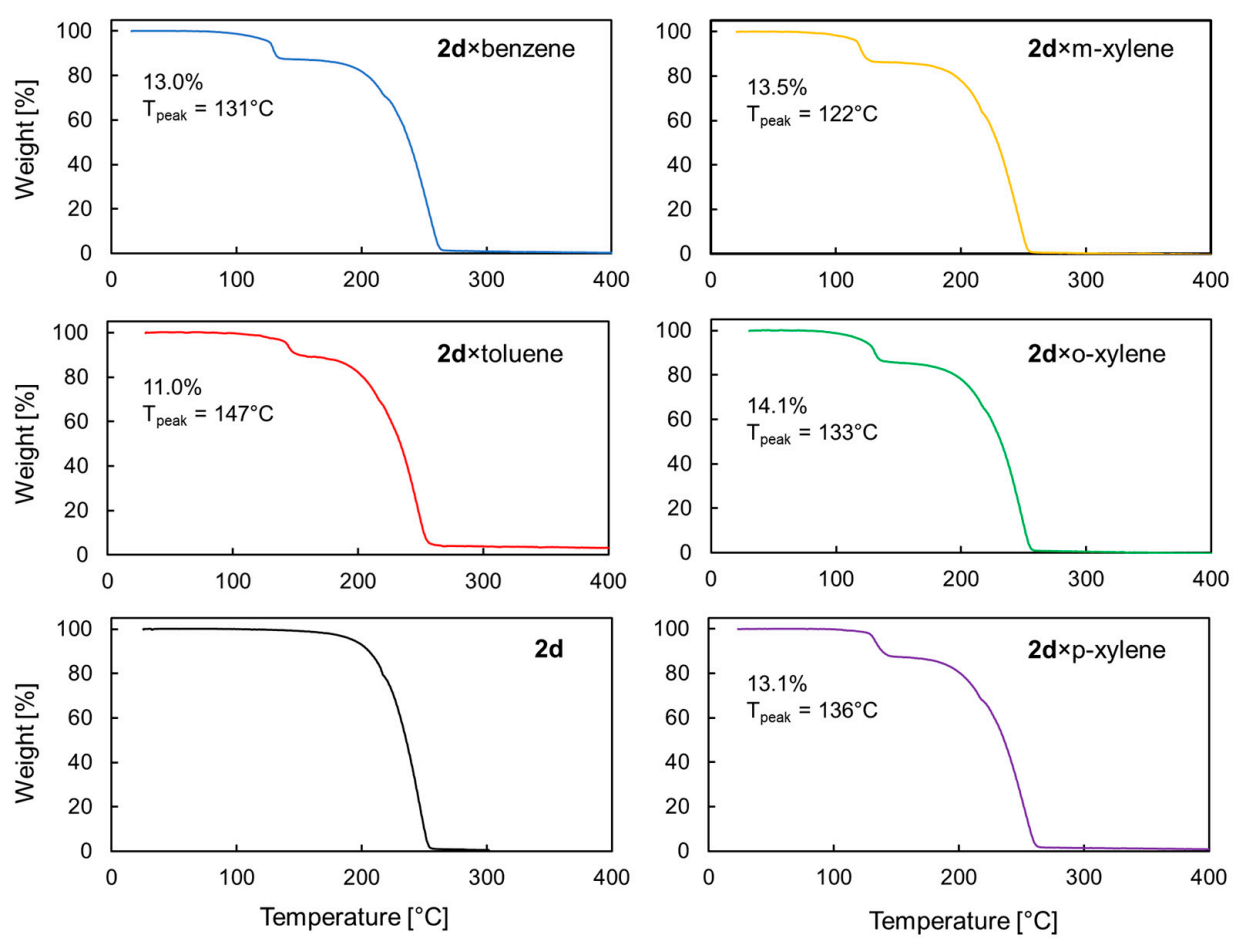
Thermogravimetric analysis of **2d** and its solvent adducts **2d** × solvent with corresponding weight loss [%] and peak temperature T_peak_ [°C] for the loss of solvent.

To study the thermal behavior of the host–guest complexes **2d** × solvent, a thermogravimetric analysis of the bulk materials was performed; the results are shown in Figure 5, Figures S30 and S31, and Table S1. Figure 5 (bottom) shows the TG curve of **2d**, revealing complete weight loss to occur at about 260 °C. This indicates that the acid–base adduct **2d** either sublimes or that the decomposition and evaporation of its individual acid and base components occur within the same temperature range. The TG graphs of all host–guest compounds exhibit a two-step weight loss. This is exemplarily shown for the solvent adducts **2d** × benzene, **2d** × toluene, **2d** × *o*-xylene, **2d** × *m*-xylene, and **2d** × *p*-xylene (Figure 5), for which the observed weight losses are consistent with the stoichiometry of the bulk materials established by ^1^H NMR spectroscopy (Table 1). After the loss of the guest, the residual material likely consists of the solid phase established for solvent-free **2d**. In this series, for **2d** × benzene and **2d** × toluene, the peak temperatures (T_peak_) associated with the liberation of the guest were found to be 131 °C and 147 °C, respectively, suggesting stronger host–guest interactions in **2d** × toluene, and consistent with the results of DSC analysis (Figures S32 and S33). For the xylene series, the peak temperatures for the loss of solvent are 122 °C (**2d** × *m*-xylene), 133 °C (**2d** × *o*-xylene), and 136 °C (**2d** × *p*-xylene). The slightly higher peak temperatures for **2d** × *o*-xylene and **2d** × *p*-xylene suggest somewhat stronger host–guest interactions compared to **2d** × *m*-xylene, which is supported by DSC measurements (Figures S34–S36).

In a related study, Hőpfl and Morales-Rojas recently disclosed the thermal properties of self-assembled host–guest complexes derived from reactions of 1,2-catechol and 3,4,5-trifluorphenylboronic acid with 4,4′-bipyridine in various aromatic hydrocarbon solvents [28]. Thermogravimetric analysis of these catechol-based host–guest complexes revealed the peak temperatures associated with the loss of solvent to be 65 °C (*m*-xylene), 71 °C (*o*-xylene), 76 °C (*p*-xylene), 121 °C (toluene), 139 °C (benzene), and 100 °C (mesitylene). Except for benzene, these values are significantly lower than what was measured for our 1,8-dihydroxy naphthalene-based host–guest complexes **2d** × solvent (Figure 5). The replacement of catechol with the 1,8-dihydroxy naphthalene ligand at the central boron appears to result in stronger π-type host–guest interactions between the 4,4′-bipyridine unit and the aromatic solvent. Unfortunately, Hőpfl and Morales-Rojas did not disclose thermodynamic data from DSC measurements for comparison [28].

Based on the results from the host–guest chemistry with *m*-, *o*-, and *p*-xylene, we wondered whether self-assembled **2d** would selectively incorporate one of the three xylene isomers. Thus, 1,8-dihydroxy naphthalene, 4,4′-bipyridine, and 3,4,5-trifluoroboronic acid were reacted in an equimolar solvent mixture of *m*-, *p*-, and *o*-xylene (1:1:1). The obtained crystalline material was collected and analyzed by ^1^H NMR spectroscopy using DMSO-D_6_ as the solvent (see supporting information for more details). The results revealed a higher selectivity of **2d** for *o*-xylene (ca. 57%), with an *m*-, *p*-, to *o*-xylene ratio of about 2:1:4, respectively, which is similar to those of the respective catechol-based host–guest complexes reported by Hőpfl and Morales-Rojas [28]. Considering the results from the TGA and DSC measurements, the fairly low uptake of *p*-xylene appears to be surprising but perhaps can be attributed to kinetic effects during the crystallization process.

**Figure 6:**
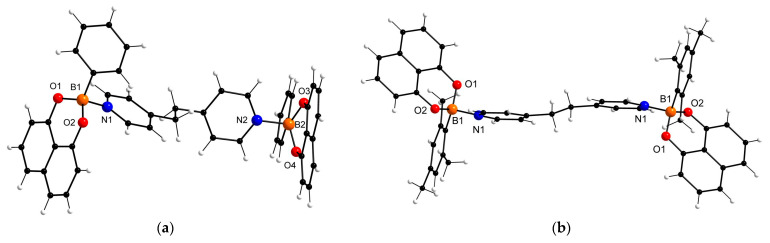
Solid-state structures of **5** and **6** (color codes: red = oxygen, orange = boron, white = hydrogen, black = carbon, blue = nitrogen). Selected bond lengths [Å] and angles [°]: (**a**) **5**, O1 B1 1.447(4), O2 C3 1.367(3), O2 B1 1.466(4), N1 B1 1.656(4), N2 B2 1.682(4), O1 B1 O2 114.4(3), O1 B1 N1 105.4(2), O2 B1 N1 104.7(2); (**b**) **6**, O1 B1 1.471(2), O2 B1 1.467(2), N1 B1 1.622(2), O2 B1 O1 112.3(1), O2 B1 N1 106.0(1), O1 B1 N1 99.7(1).

Finally, to further broaden the scope of the host–guest approach, the modified bipyridine linker 1,2-bis(4-pyridyl)ethane was employed in reactions with 1,8-dihydroxy naphthalene and various boronic acids. Unfortunately, in none of the cases could host–guest complexes with aromatic hydrocarbon solvents be crystallized and structurally determined. Nonetheless, single crystals of the solvent-free double-tweezers **5** and **6** could be obtained from acetone solutions and were characterized by single-crystal X-ray crystallography (Figure 6a,b).

## 3. Conclusions

We have demonstrated the ability of 1,8-dihydroxy naphthalene to self-assemble with various aryl boronic acids and 4,4′-bipyridine as a linker to give double-tweezer and H-shaped Lewis acid–base adducts in very good isolated yields by means of N→B bond formation. In addition, a series of host–guest complexes, **2d** × solvent, was prepared via reactions of 4,4′-bipyridine, 1,8-dihydroxy naphthalene, and 3,4,5-trifluorophenyl boronic acid with various aromatic hydrocarbons. SCXRD analysis of these solvent adducts revealed host–guest interactions to primarily occur via π···π contacts between the 4,4′-bipyridyl linker and the aromatic solvents to give 1:1 and 1:2 host–guest complexes, which was confirmed by ^1^H NMR spectroscopic analysis of the bulk samples. TGA measurements of the bulk samples showed well-defined weight losses at peak temperatures ranging from 122 to 147 °C, which in every case could be attributed to the complete loss of solvent (guest). Self-assembled Lewis acid–base adduct **2d**, when recrystallized from an equimolar mixture of *m*-, *p*-, and *o*-xylene (1:1:1), preferentially binds to *o*-xylene, generating **2d** × *o*-xylene as the major component, similar to what was reported for structurally related catechol-based host–guest complexes [28]. Collectively, the present contribution demonstrates the effectiveness of 1,8-dihydroxy naphthalene in constructing supramolecular structures via dative N-B bond formation.

## Figures and Tables

**Table 1:** Molar **2d**/solvent ratios of **2d** × solvent from SCXRD and ^1^H NMR data.

2d × Solvent	2d/Solvent Ratio (from X-ray Data)	2d/Solvent Ratio (from ^1^H NMR Data) ^1^
**2d** × benzene	2:3	1:1.5
**2d** × toluene	1:1	1:1
**2d** × *o*-xylene	1:2	1:1
**2d** × *m*-xylene ^2^	3:6 ^3^ and 1:1	1:1
**2d** × *p*-xylene	1:1	1:1
**2d** × mesitylene	1:2 ^4^	1:2.66
**2d** × aniline	2:3	1:1.5

^1^ The isolated crystalline materials were dried in air for 24 h; ^2^ Two crystallographically distinct crystals were found; ^3^ Crystal contains two additional unbound *m*-xylene molecules; ^4^ Crystal contains two molecules of **2d** and two molecules of unbound mesitylene.

## Data Availability

Not applicable.

## Supplementary Materials

The following supporting information can be downloaded at: https://www.mdpi.com/article/10.3390/molecules28145394/s1. Figure S1: ^1^H NMR spectrum of **2c** (acetone-d_6_); Figure S2: ^13^C NMR spectrum of **2c** (acetone-d_6_); Figure S3: ^11^B NMR spectrum of **2c** (acetone-d_6_); Figure S4: ^11^B NMR spectrum of **2c** (DMSO-d_6_); Figure S5: ^1^H NMR spectrum of **2c** × benzene in DMSO-d_6_; Figure S6: ^1^H NMR spectrum **2d** (acetone-d_6_); Figure S7: ^13^C NMR spectrum of **2d** (acetone-d_6_); Figure S8: ^19^F NMR spectrum of **2d** (acetone-d_6_); Figure S9: ^11^B NMR spectrum of **2d** (acetone-d_6_); Figure S10: ^1^H NMR spectrum of 4,4-bipyridinium-bis(1,8-naphthalenediolato)borate (DMSO-d_6_); Figure S11: ^11^B NMR spectrum of 4,4-bipyridinium-bis(1,8-naphthalenediolato)borate (DMSO-d_6_); Figure S12: ^13^C NMR spectrum of 4,4-bipyridinium-bis(1,8-naphthalenediolato)borate (DMSO-d_6_); Figure S13: ^13^C NMR-DEPT spectrum of 4,4-bipyridinium-bis(1,8-naphthalenediolato) borate (DMSO-d_6_); Figure S14: ^1^H NMR spectrum of 1,4-benzene-diboronic-acid-1,8-naphthalenediolate ester in DMSO-d_6_; Figure S15: ^13^C NMR spectrum of 1,4-benzene-diboronic-acid-1,8-naphthalenediolate ester in DMSO-d_6_; Figure S16: ^11^B NMR spectrum of 1,4-benzene-diboronic-acid-1,8-naphthalenediolate ester in DMSO-d_6_; Figure S17: ^11^B NMR spectrum of 1,4-benzene-diboronic-acid-1,8-naphthalenediolate ester in acetone-d_6_; Figure S18: ^1^H NMR spectrum of **4** in DMSO-d_6_; Figure S19: ^1^H NMR spectrum of **2d** × benzene in acetone-d_6_; Figure S20: ^1^H NMR spectrum of **2d** × toluene in DMSO-d_6_; Figure S21: ^1^H NMR spectrum of **2d** × *m*-xylene in DMSO-d_6_; Figure S22: ^1^H NMR spectrum of **2d** × *p*-xylene in DMSO-d_6_; Figure S23: ^1^H NMR spectrum of **2d** × *o*-xylene in DMSO-d_6_; Figure S24: ^1^H NMR spectrum of **2d** × mesitylene in DMSO-d_6_; Figure S25: ^1^H NMR spectrum of **2d** × aniline in DMSO-d_6_; Figure S26: ^1^H NMR spectrum (DMSO-d_6_) of adduct **2d** × xylene isolated from a 1:1:1 mixture of *m*-, *p*-, and *o*-xylene; Figure S27: Aliphatic region of the ^1^H NMR spectrum (DMSO-d_6_) of adduct **2d** × xylene isolated from a 1:1:1 mixture of *m*-, *p*-, and *o*-xylene; Figure S28: ^1^H NMR spectrum (DMSO-d_6_) of **2d** × *m*-xylene after soaking in *o*-xylene for 24 h; Figure S29: ^1^H NMR spectrum (DMSO-d_6_) of **2d** × *p*-xylene after soaking in *o*-xylene for 24 h; Figure S30: Thermogravimetric analysis of **2d** × mesitylene with weight loss [%] and peak temperature T_peak_ [°C] for the loss of solvent; Figure S31: Thermogravimetric analysis of **2d** × aniline with weight loss [%] and peak temperature T_peak_ [°C] for the loss of solvent; Figure S32: DSC analysis of **2d** × benzene for the loss of solvent; Figure S33: DSC analysis of **2d** × toluene for the loss of solvent; Figure S34: DSC analysis of **2d** × p-xylene for the loss of solvent; Figure S35: DSC analysis of **2d** × o-xylene for the loss of solvent; Figure S36: DSC analysis of **2d** × m-xylene for the loss of solvent; Table S1: Selected information from the TGA analysis for **2d** × solvent; Table S2: Crystal data and structure refinement for **2d** × toluene; Table S3: Crystal data and structure refinement for **6**; Table S4: Crystal data and structure refinement for **2b**; Table S5: Crystal data and structure refinement for **2d** × benzene; Table S6: Crystal data and structure refinement for **2d** × *p*-xylene; Table S7: Crystal data and structure refinement for **2d** × *m*-xylene; Table S8: Crystal data and structure refinement for **2d** × aniline; Table S9: Crystal data and structure refinement for **5**; Table S10: Crystal data and structure refinement for **2d**; Table S11: Crystal data and structure refinement for **3**; Table S12: Crystal data and structure refinement for **2d** × *m*-xylene; Table S13: Crystal data and structure refinement for **2c**; Table S14: Crystal data and structure refinement for **2a**; Table S15: Crystal data and structure refinement for **2d** × mesitylene; Table S16: Crystal data and structure refinement for **2d** × *o*-xylene; Table S17: Crystal data and structure refinement for **4**. References [31,32,33,34,35,36,37,38] are cited in the supplementary materials.
