# Supplementary material for: 1,8-Dihydroxy Naphthalene—A New Building Block for the Self-Assembly with Boronic Acids and 4,4′-Bipyridine to Stable Host–Guest Complexes with Aromatic Hydrocarbons
Source: Molecules. 2023 Jul 14;28(14):5394. doi: 10.3390/molecules28145394 (PMC10385103; doi:10.3390/molecules28145394)
Supplement: Supplementary file 1 [file molecules-28-05394-s001.zip › molecules-2402269-supplementary.pdf]

Supporting Information

1,8-Dihydroxy Naphthalene – A New Building Block for the Self-Assembly with Boronic Acids and 4,4'-Bipyridine to Stable Host-Guest Complexes with Aromatic Hydrocarbons

C. P. Manankandayalage, D. K. Unruh, R. Perry, C. Krempner

Texas Tech University, Department of Chemistry & Biochemistry, Box 41061, Lubbock, Texas, 79409-1061. Clemens.krempner@ttu.edu

1. General Remarks

Solvents were purchased from chemical vendors and used as received. 4,4'-bipyridine (Strem), trans-1,2-di(4-pyridyl)ethylene (Sigma Aldrich), 1,2-bis(4-pyridyl)ethane (Sigma Aldrich), 1,8-dihydroxynaphthalene (Accela), phenylboronic acid (Chem-Impex), 3,4,5-trifluorophenylboronic acid (Frontier Scientific), 2,4,6-trimethylphenylboronic acid (Matrix Scientifics), pentafluorophenylboronic acid (BTC chemicals), and 2,6-dichlorophenylboronic acid (Combi Blocks), 1,4-phenylene-bisboronic acid (Combi Blocks) and 1,3-phenylene-bisboronic acid (Combi Blocks) were purchased from commercial sources and used without further purification.

The boronic acid esters **1a-e** were prepared according to a published literature procedure [Manankandayalage, C.P.; Unruh, D.K.; Krempner, C. *Dalton Trans.* **2020**, *49*, 4834-4842.].

The ^{1}H-, ^{13}C-, ^{11}B- ^{19}F-NMR spectra were obtained from a JOEL ECS 400. All measurements, unless noted otherwise, were carried out at 298 K and NMR chemical shifts were given in ppm. The ^{11}B NMR spectra referenced to H_3BO_3 in D_2O (δ = 36 ppm). The ^{19}F NMR spectrum was referenced to $C_6H_5CF_3$ in C_6D_6 (δ = 62.3 ppm). The ^{1}H-NMR spectra were referenced to the residual protonated solvent for ^{1}H. The ^{13}C NMR spectra were referenced to the deuterated solvent peaks. The following abbreviations were used to describe peak multiplicities in the reported NMR spectroscopic data: "s" for singlet, "d" for doublet, "t" for triplet, "q" for quartet, "sept" for septet, "m" for multiplet and "br" for broadened resonances. Elemental analyses were performed using a Perkin Elmer 2400 Series II CHNS/O Analyzer.

Thermogravimetric analyses were performed on a Shimadzu DTG-60H instrument with approximately 3-5 mg of a solid sample at a heating rate of 10 °C/min within the temperature range of 25−400 °C using a current of nitrogen as inert gas purge (100 mL/min). Differential scanning calorimetry analyses were performed on a Shimadzu DSC-60Plus with approximately 3-5 mg of a solid sample at a heating rate of 10 °C/min within the temperature range of 25−200 °C using a current of nitrogen as inert gas purge (100 mL/min).

2. Experimental

2.1. NMR spectroscopic investigations of the reaction of boronic esters with 4,4'-bipyridine

A standard NMR tube was charged with two equivalents of the respective ester, one equivalent of 4,4'-bipyridine and 0.5 mL of acetone-D_6. The resulting solutions were analyzed by ^{11}B-NMR spectroscopy.

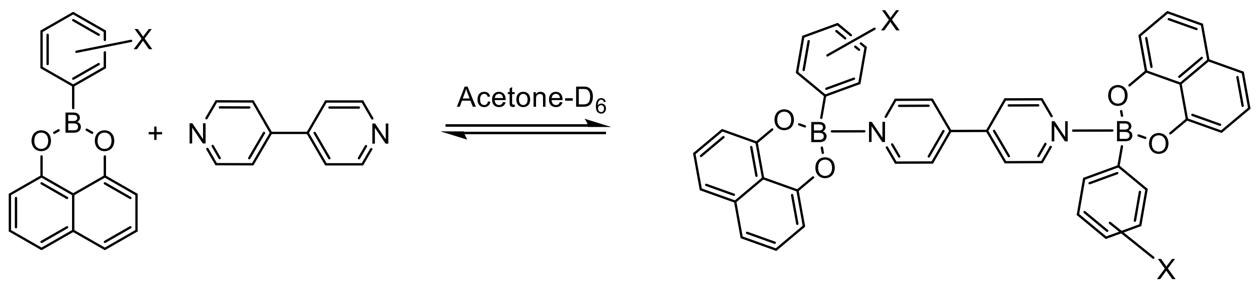

aryl-X = C_6H_5, 3,4,5-$C_6F_3H_2$, C_6F_5, 2,6-$C_6Cl_2H_3$, 2,4,6-$(CH_3)_3$-C_6H_2

2.2. Synthesis of **2c.**

Phenylboronic acid (7.3 mg, 0.06 mmol), 1,8-dihydroxy naphthalene (9.6 mg, 0.06 mmol), and 4,4'-bipyridine (4.7 mg, 0.03 mmol) were dissolved in acetone (1 mL) in a 4 mL scintillation vial. The solution was gently heated until all the starting material dissolved. The mixture was allowed to cool to room temperature and the pure product was obtained by decantation. Bright orange crystalline solid (16 mg, 80%). M.p. 216-217 °C. ^{1}H NMR (400 MHz, acetone-d_6): δ = 8.95 (d, $^3\boldsymbol{J}_{H\text{-}H}$ = 8 Hz, CH-bipyridine, 4 H), 7.92 (d, $^3\boldsymbol{J}_{H\text{-}H}$ = 8 Hz, CH-bipyridine, 4 H), 7.79 (d, $^3\boldsymbol{J}_{H\text{-}H}$ = 8 Hz, CH-bipyridine, 4 H), 7.24-7.31 (m, CH, 14 H), 6.88 (d, $^3\boldsymbol{J}_{H\text{-}H}$ = 8 Hz, CH, 4 H), ^{13}C NMR (100.6 MHz, DMSO-d_6): δ = 108.1, 116.8, 117.2, 123.1, 127.1, 127.2, 127.6, 131.2, 135.2, 147.8, 151.9 ppm. Note: The ipso carbon bound to boron could not be detected in the ^{13}C NMR. ^{11}B NMR (128.4 MHz, DMSO-d_6): δ = 6.9 ppm. ^{11}B NMR (128.4 MHz, acetone-d_6): δ = 11.6 ppm. Anal. Calc. for $C_{42}H_{30}B_2N_2O_4$ (648.33): C, 77.81; H, 4.66; N, 4.32. Found: C, 77.74, H, 4.57, 4.07.

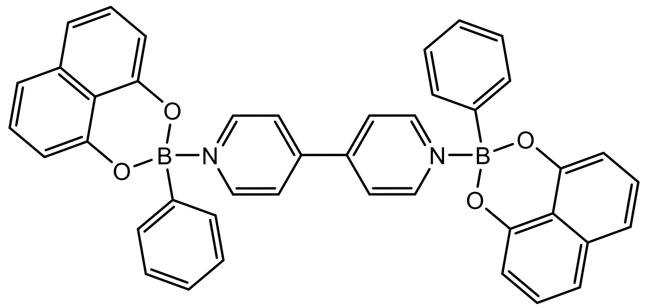

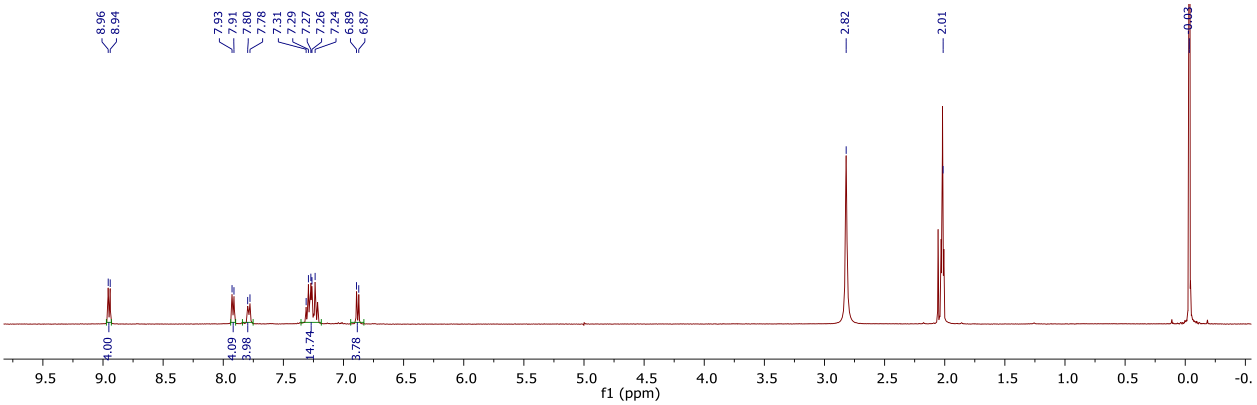

Figure S1. ^{1}H NMR spectrum of **2c** (acetone-d$_6$).

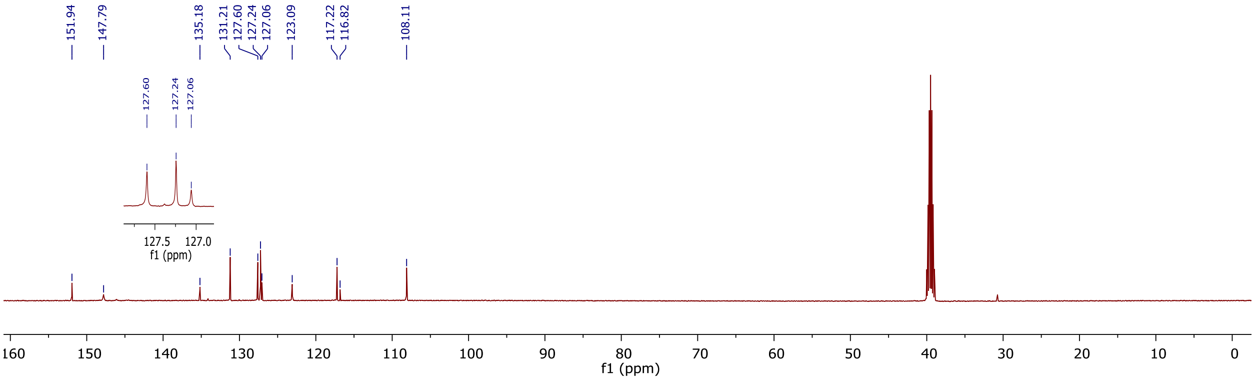

Figure S2. ^{13}C NMR spectrum of **2c** (acetone-d$_6$).

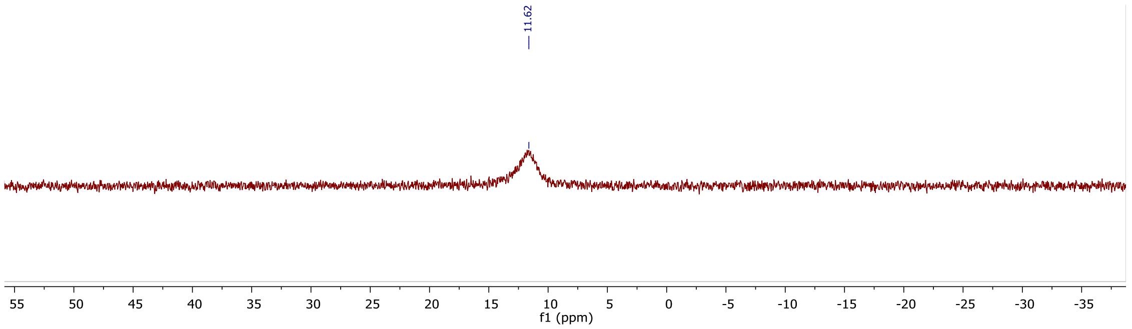

Figure S3. ^{11}B NMR spectrum of **2c** (acetone-d$_6$).

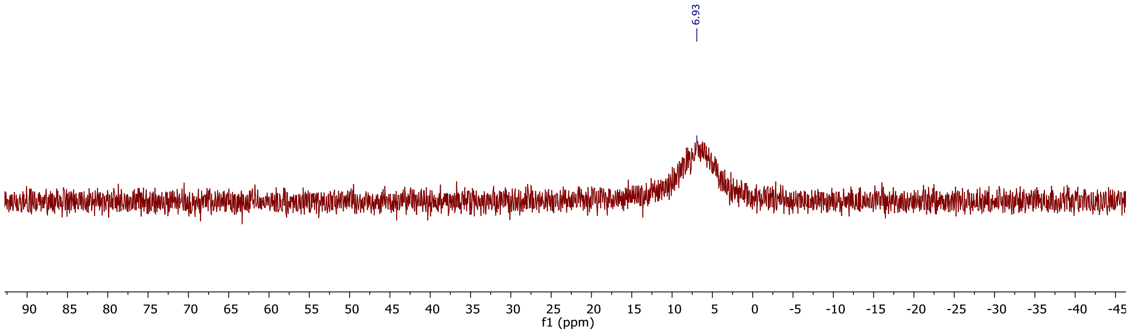

Figure S4. ^{11}B NMR spectrum of **2c** (DMSO-d$_6$).

2.3. Synthesis of **2c**×benzene.

Phenylboronic acid (38 mg, 0.3 mmol), 1,8-dihydroxy naphthalene (50 mg, 0.3 mmol), 4,4' bipyridine (24 mg, 0.15 mmol), and 4 mL of benzene were added to a 20 mL scintillation vial. The mixture was heated until all the starting material dissolved. Upon cooling to room temperature yellow single crystals formed, which were collected by decantation. The crystals were washed once with benzene, twice with hexanes, and dried in vacuo for only 10 min to avoid further loss of benzene. Yield 100 mg, 83 %. M.p. 216-217 °C. ^{1}H NMR (400 MHz, DMSO-d$_6$): δ = 8.85 (d, $^{3}\boldsymbol{J}_{H\text{-}H}$ = 4 Hz, CH-bipyridine, 4 H), 7.96 (d, $^{3}\boldsymbol{J}_{H\text{-}H}$ = 4 Hz, CH-bipyridine, 4 H), 7.65 (d, $^{3}\boldsymbol{J}_{H\text{-}H}$ = 4 Hz, CH-naphthyl, 4 H), 7.40 (s, CH-benzene, 10 H), 7.34-7.20 (m, 14 H), 6.52 (d, $^{3}\boldsymbol{J}_{H\text{-}H}$ = 4 Hz, CH-naphthyl, 4 H) ppm.

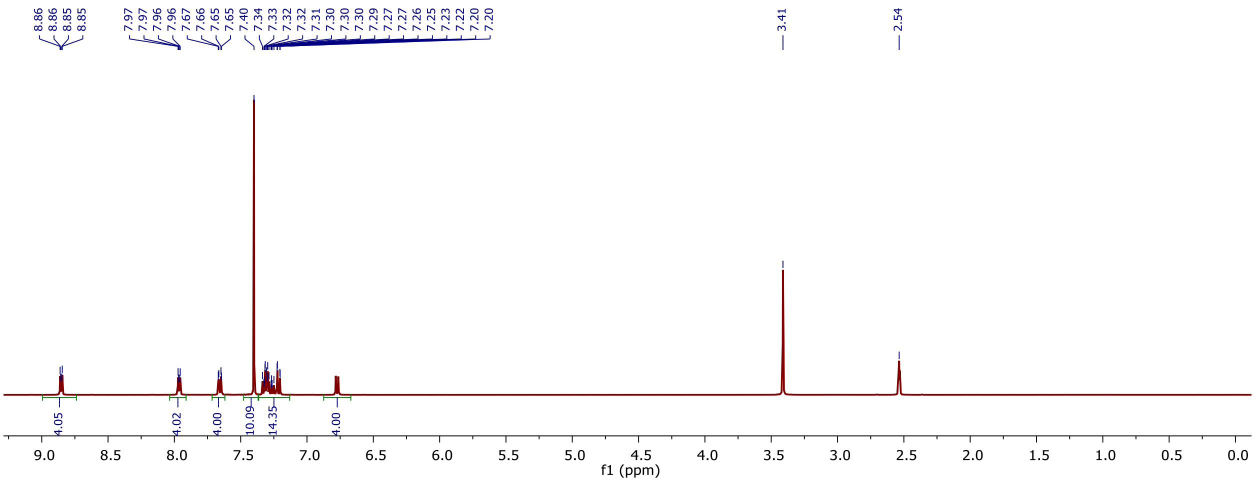

Figure S5. ^{1}H NMR spectrum of **2c**×benzene in DMSO-d$_6$.

2.4. Synthesis of **2d**

3,4,5-trifluorophenyl boronic acid (11 mg, 0.06 mmol), 1,8-dihydroxy naphthalene (10 mg, 0.06 mmol), and 4,4'-bipyridine (5 mg, 0.03 mmol) were dissolved in acetone (1.0 mL) in a 4 mL scintillation vial. The solution was gently heated until all the starting material dissolved. The mixture was allowed to cool to room temperature and the pure product was obtained as an orange microcrystalline solid. Crystals of **2d** suitable for single-crystal X-ray analysis were

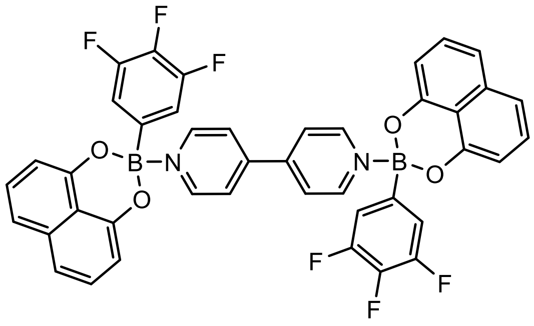

grown from acetone solutions of the reaction mixture. Yield 20 mg (90%). M.p. 215-216 °C. ^{1}H NMR (400 MHz, acetone-d$_6$): δ = 9.18 (d, $^{3}\boldsymbol{J}_{H-H}$ = 4 Hz, CH-bipyridine, 4 H), 8.16 (d, $^{3}\boldsymbol{J}_{H-H}$ = 4 Hz, CH-bipyridine, 4 H), 7.52 (t, $^{3}\boldsymbol{J}_{H-H}$ = 8 Hz, CH-arom., 4 H), 7.33 (t, $^{3}\boldsymbol{J}_{H-H}$ = 8 Hz, CH, 4 H), 7.26 (d, $^{3}\boldsymbol{J}_{H-H}$ = 8 Hz, CH, 4 H), 6.93 (d, $^{3}\boldsymbol{J}_{H-H}$ = 8 Hz, CH-naphthyl, 4 H) ppm. ^{13}C NMR (100.6 MHz, acetone-d$_6$): δ = 151.7 (ddd, $^{1}J_{C-F}$ = 199 Hz, $^{2}\boldsymbol{J}_{C-F}$ = 80 Hz, $^{3}\boldsymbol{J}_{C-F}$ = 2 Hz, meta-CF), 150.7 (C-quart), 149.5 (C-quart), 146.6 (C-quart), 139.8 (dt, $^{1}\boldsymbol{J}_{C-F}$ = 199, $^{2}\boldsymbol{J}_{C-F}$ = 13 Hz, para-CF), 136.6 (C-quart), 128.4 (CH-naphthyl), 125.8 (C-quart), 119.3 (CH-naphthyl), 118.2 (C-quart), 115.4 (dd, $^{2}\boldsymbol{J}_{C-F}$ = 12 Hz, $^{3}\boldsymbol{J}_{C-F}$ = 3 Hz, CH-phenyl), 109.8 (CH-naphthyl) ppm. ^{11}B NMR (128.4 MHz, acetone-d$_6$): δ = 5.2 ppm. ^{19}F NMR (128.4 MHz, acetone-d$_6$): δ = -137.5, -164.8 ppm Anal. Calc. for $C_{42}H_{24}B_2F_6N_2O_4$ (756.27): C, 66.70; H, 3.20, N, 3.70; Found: C, 66.47, H, 2.98, N, 3.70.

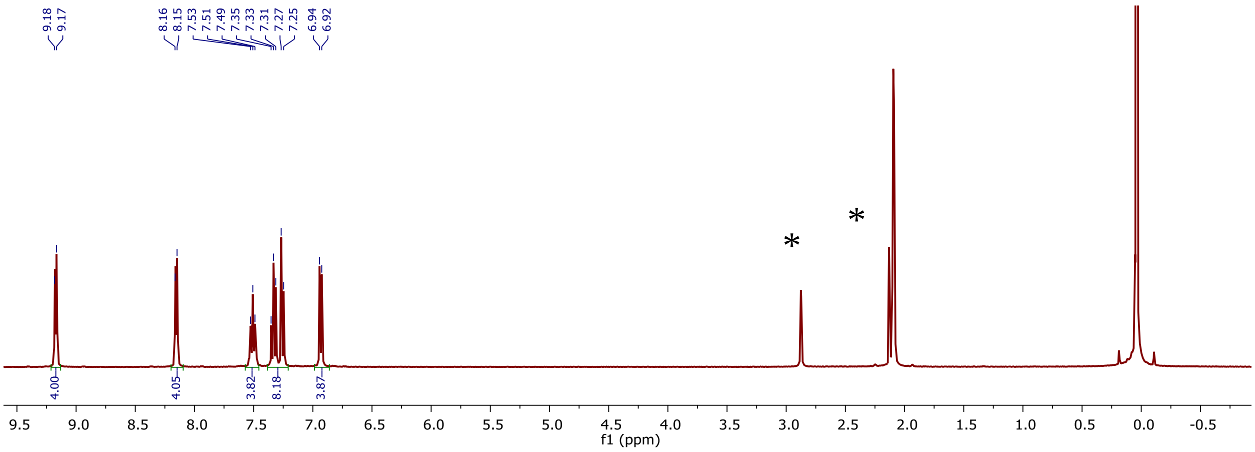

Figure S6. ^{1}H NMR spectrum **2d** (acetone-d$_6$). The water signal from the solvent and acetone is labeled with asterisks.

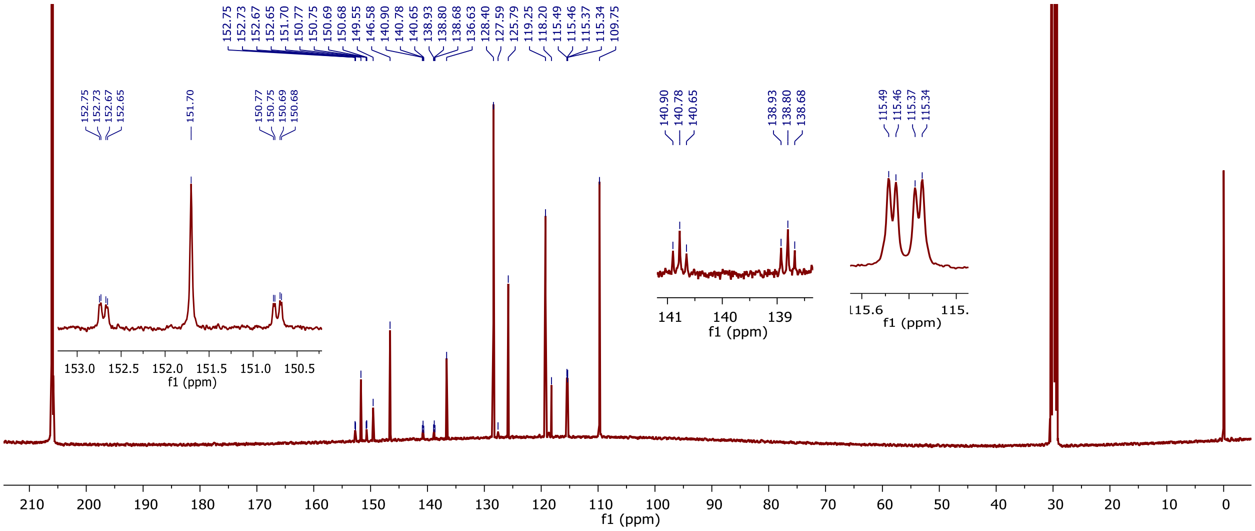

Figure S7. ^{13}C NMR spectrum of **2d** (acetone-d$_6$).

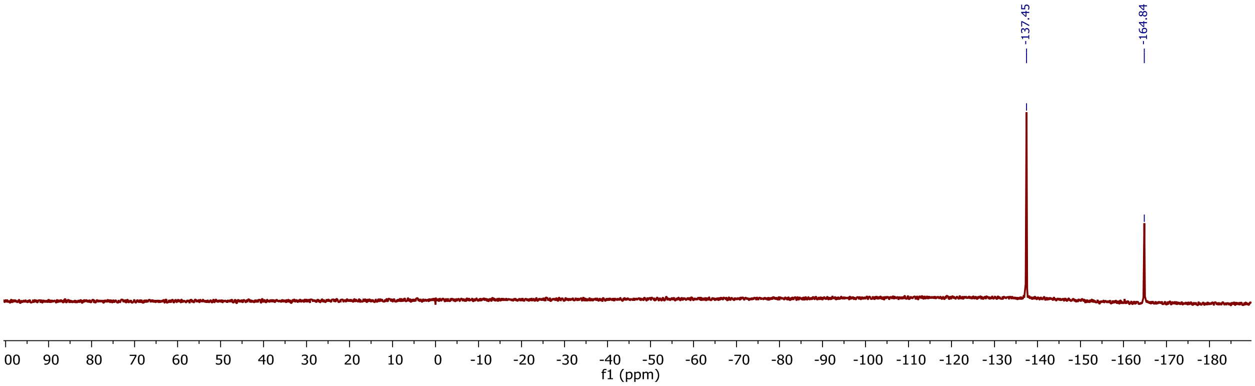

Figure S8. ^{19}F NMR spectrum of **2d** (acetone-d$_6$).

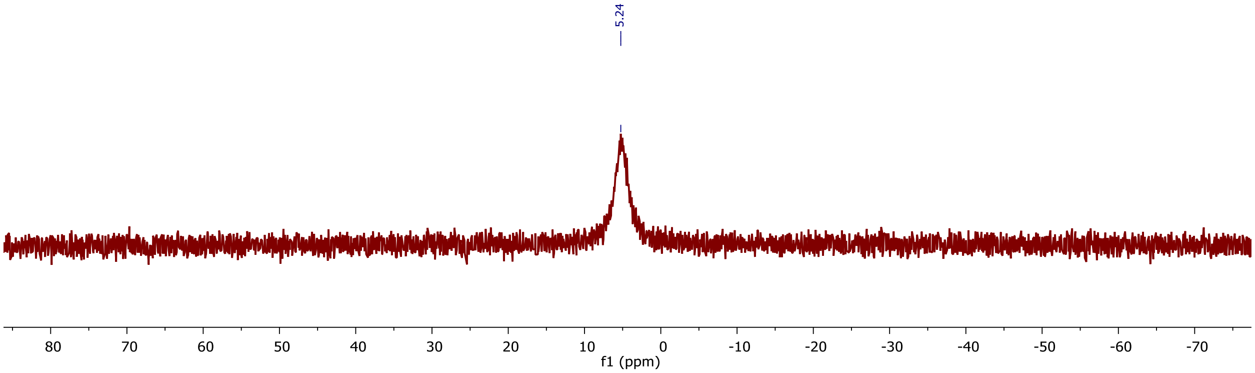

Figure S9. ^{11}B NMR spectrum of **2d** (acetone-d$_6$).

2.5. Reaction of pentafluorophenylboronic acid with 4,4'-bipyridine and 1,8-dihydroxy naphthalene – Formation of spirocyclic 4,4'-bipyridinium-bis(1,8-naphthalenediolato) borate

An NMR tube was charged with pentafluorophenyl boronic acid (13 mg, 0.068 mmol), 1,8-dihydroxy naphthalene (11 mg, 0.068 mmol), and 4,4'-bipyridine (5.4 mg, 0.034 mmol) and acetone-d_6. The reaction mixture was gently heated until all reactants dissolved and

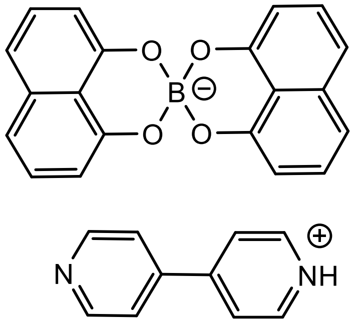

subsequently analyzed by ^{1}H and ^{11}B NMR spectroscopy. According to the NMR data, a secondary product formation was observed. The reaction mixture was monitored for over a week. During that time dark red crystals of the title compound formed, which were collected, and dried in vacuo. ^{1}H NMR (400 MHz, DMSO-d_6): δ = 8.96 (d, $^{3}\boldsymbol{J}_{H\text{-}H}$ = 8 Hz, CH-bipyridine, 4 H), 8.22 (d, $^{3}\boldsymbol{J}_{H\text{-}H}$ = 8 Hz, CH-bipyridine, 4 H), 7.20 (t, $^{3}\boldsymbol{J}_{H\text{-}H}$ = 8 Hz, 4 H), 7.09 (d, $^{3}\boldsymbol{J}_{H\text{-}H}$ = 8 Hz, CH, 4 H), 6.41 (d, $^{3}\boldsymbol{J}_{H\text{-}H}$ = 8 Hz, CH, 4 H) ppm. ^{13}C NMR (100.6 MHz, DMSO-d_6): δ = 106.7 (CH-bipyridine), 115.4 (CH-naphthyl), 116.5 (C-quart), 122.9 (CH-naphthyl), 127.4 (CH-naphthyl), 135.5 (C-quart), 146.8 (C-quart), 147.7 (CH-bipyridine), 154.9 ppm. ^{11}B NMR (128.4 MHz, DMSO-d_6): δ = 0.5 ppm.

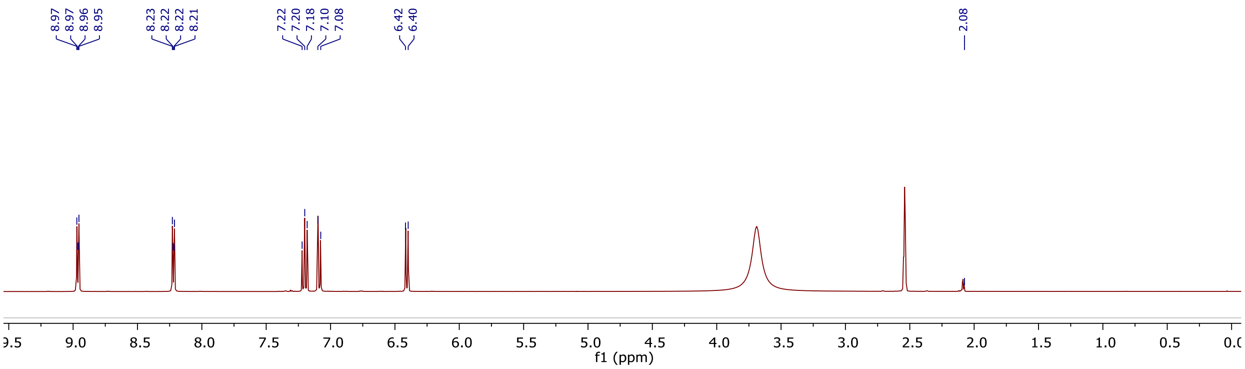

Figure S10. ^{1}H NMR spectrum of 4,4-bipyridinium-bis(1,8-naphthalenediolato)borate (DMSO-d_6).

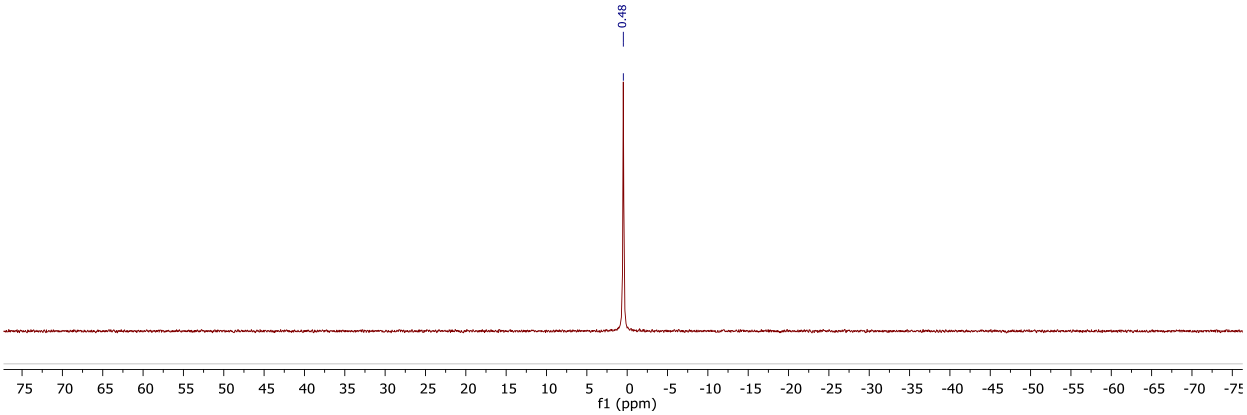

Figure S11. ^{11}B NMR spectrum of 4,4-bipyridinium-bis(1,8-naphthalenediolato)borate (DMSO-d_6).

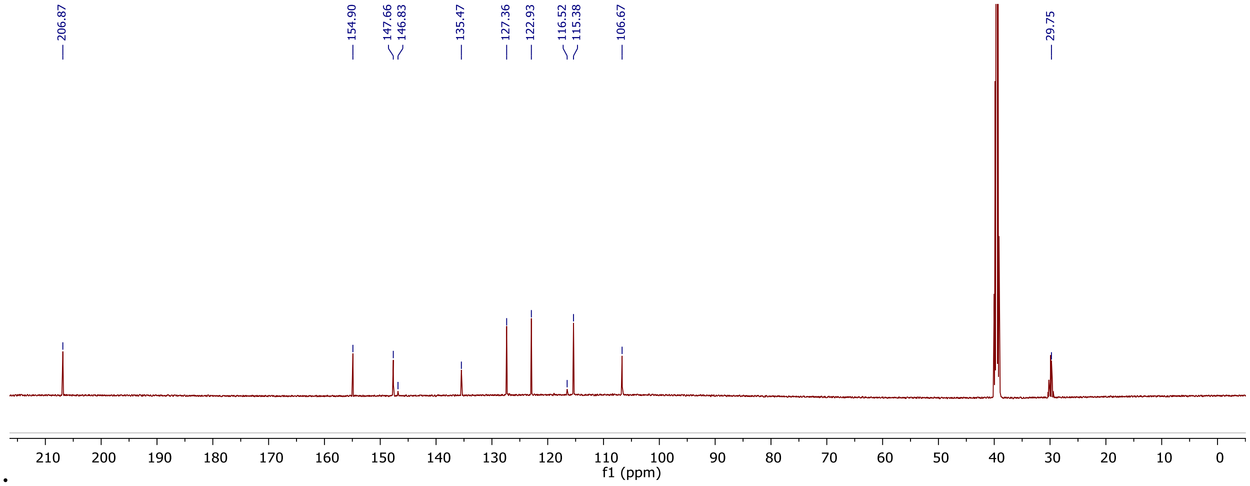

.

Figure S12. ^{13}C NMR spectrum of 4,4-bipyridinium-bis(1,8-naphthalenediolato)borate (DMSO-d_6).

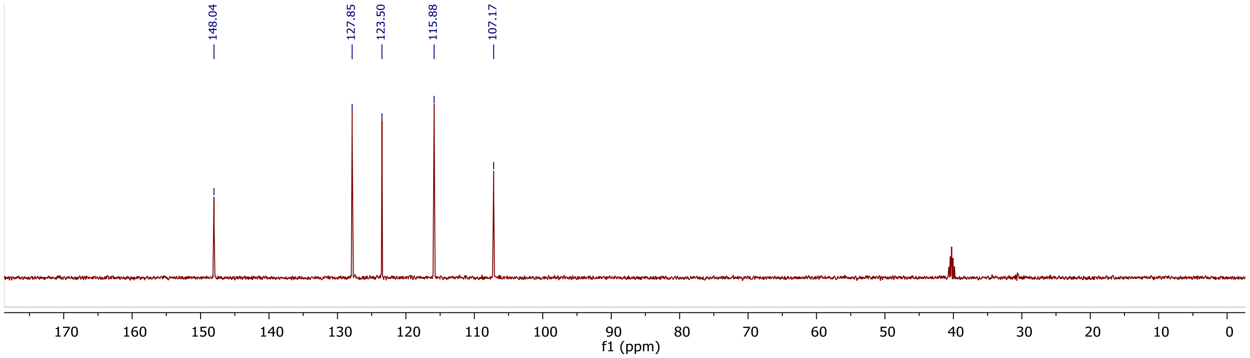

Figure S13. ^{13}C NMR-DEPT spectrum of 4,4-bipyridinium-bis(1,8-naphthalenediolato) borate (DMSO-d_6).

2.6. Synthesis of 1,4-benzene-diboronic-acid-1,8-naphthalenediolate ester

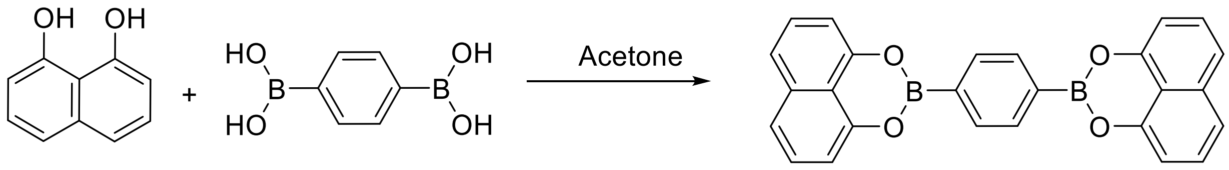

A 20 mL scintillation vial was charged with 1,8-dihydroxy naphthalene (0.2 g, 1.25 mmol), 1,4-benzene-diboronic acid (0.1 g, 1.25 mmol), and 10 mL of acetone. Upon stirring the mixture overnight, white microcrystals formed, which were collected by decantation, washed twice with acetone and dried in vacuo. Yield 0.27 g (75%). M.p. >260 °C. ^{1}H NMR (400 MHz, DMSO-d_6): δ = 7.62 (s, CH-phenylene, 4 H), 7.32 (t, $^{3}J_{H-H}$ = 8 Hz, CH-naphthalene, 4 H), 7.21 (d, $^{3}J_{H-H}$ = 8 Hz, CH-naphthalene, 4 H), 6.70 (d, $^{3}J_{H-H}$ = 8 Hz, CH-naphthalene, 4 H) ppm. ^{13}C NMR (100.6 MHz, DMSO-d_6): δ = 152.1 (C-quart), 135.3 (CH), 130.9 (CH), 127.5 (CH), 117.2 (C-quart), 107.7 (CH), 106.9 (C-quart) ppm. Note: The ipso carbon bound to boron could not be detected in the ^{13}C NMR spectrum. ^{11}B NMR (128.4 MHz, DMSO-d_6): δ = 10.5 ppm. ^{11}B NMR (128.4 MHz, acetone-d_6): δ = 26.5 ppm.

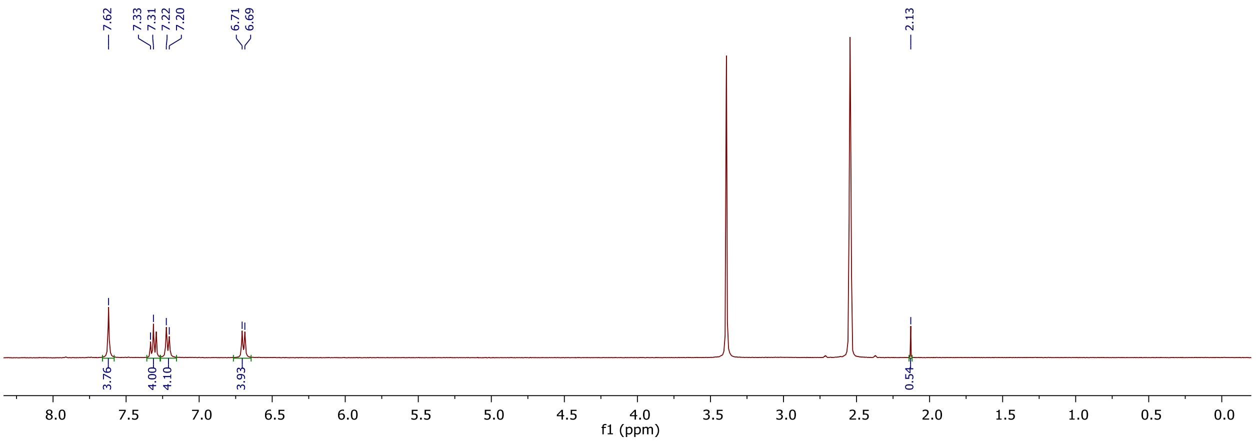

Figure S14. ^{1}H NMR spectrum of 1,4-benzene-diboronic-acid-1,8-naphthalenediolate ester in DMSO-d_6.

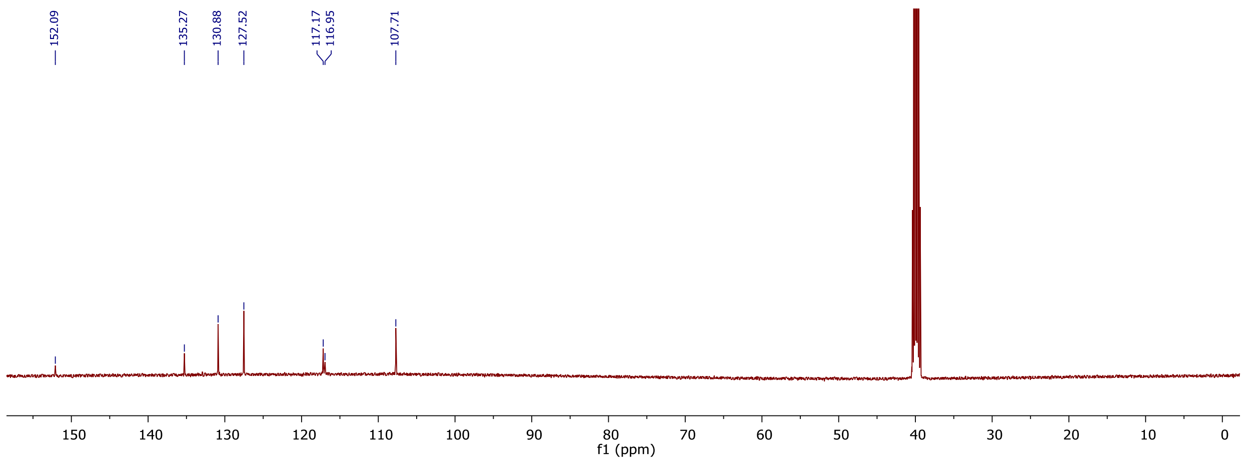

Figure S15. ^{13}C NMR spectrum of 1,4-benzene-diboronic-acid-1,8-naphthalenediolate ester in DMSO-d_6.

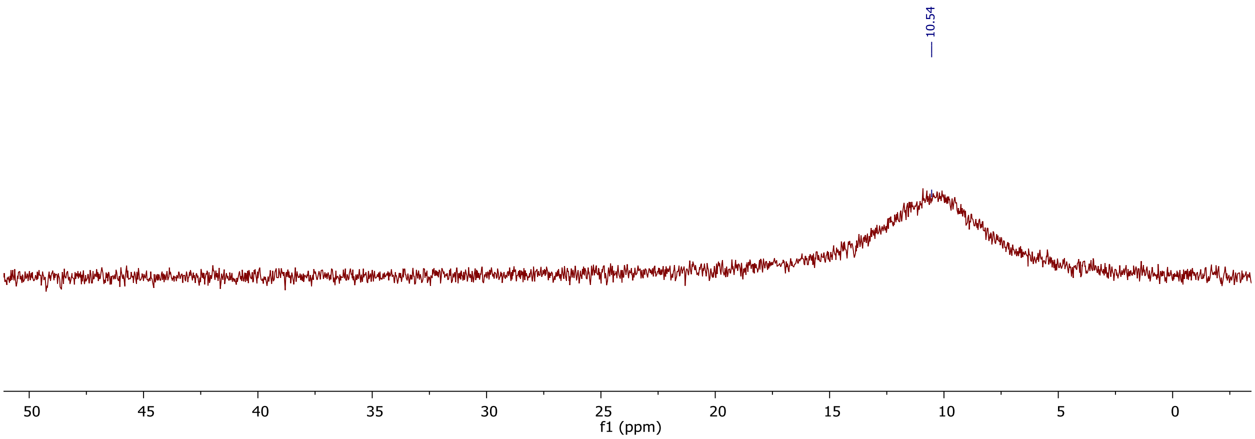

Figure S16. ^{11}B NMR spectrum of 1,4-benzene-diboronic-acid-1,8-naphthalenediolate ester in DMSO-d_6.

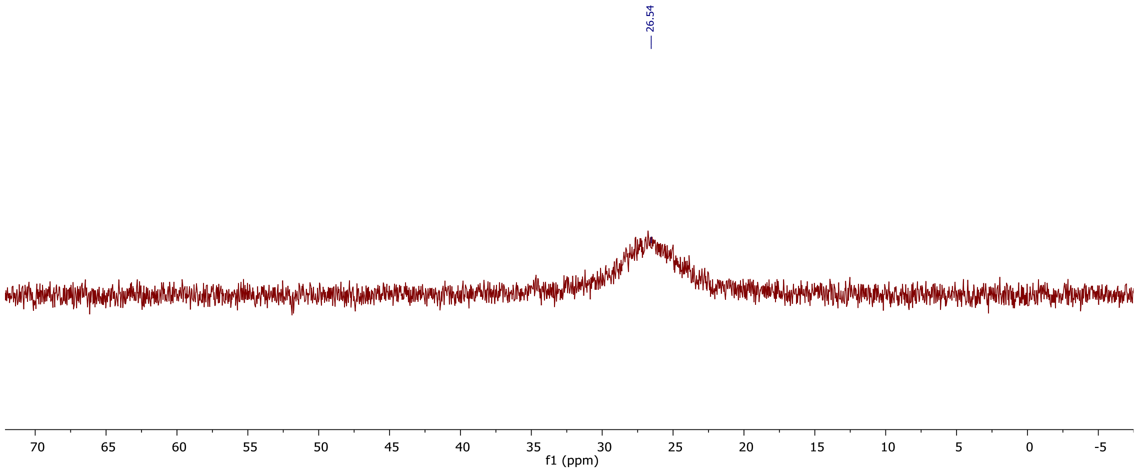

Figure S17. ^{11}B NMR spectrum of 1,4-benzene-diboronic-acid-1,8-naphthalenediolate ester in acetone-d_6.

2.7. Formation of **3**

A mixture of 1,4-phenylene bis boronic acid (30 mg, 0.18 mmol), 1,8-dihydroxy naphthalene (58 mg, 0.36 mg), trans-1,2-bis(4-pyridyl)ethylene (33 mg, 0.18 mmol) and 5 mL of acetone was stirred overnight at room temperature. The obtained microcrystals were washed twice with acetone and dried in a vacuum. Due to its extremely poor solubility in organic solvents, NMR spectroscopic analysis of **3** was not possible. Single crystals of **3** suitable for X-ray analysis were grown from dilute DMSO solutions.

2.8. Synthesis of **4**

A mixture of 1,3-phenylene bis boronic acid (22 mg, 0.13 mmol), 1,8-dihydroxy naphthalene (41 mg, 0.26 mg), 4,4'-bipyridine (20 mg, 0.13 mmol) and 5 mL of acetone was stirred overnight at room temperature. The obtained greenish-yellow powder was washed twice with acetone, and dried in vacuo. Single crystals suitable for X-ray analysis were grown from dilute acetone solutions. Yield 70 mg (94 %). ^{1}H NMR (400 MHz, DMSO-d_6): δ = 8.70 (d, $^{3}J_{H\text{-}H}$ = 5 Hz, CH-bipyridine, 8 H), 7.93 (s, 1,3-phenylene, 2 H), 7.80 (d, $^{3}\boldsymbol{J}_{H\text{-}H}$ = 5 Hz, CH-bipyridine, 8 H), 7.47 (d, $^{3}\boldsymbol{J}_{H\text{-}H}$ = 7 Hz, 1,3-phenylene, 4 H), 7.23 (t, $^{3}\boldsymbol{J}_{H\text{-}H}$ = 8 Hz, naphthalene, 8 H) 7.17 (tr, $^{3}\boldsymbol{J}_{H\text{-}H}$ = 7 Hz, 1,3-phenylene, 2 H), 7.11 (d, $^{3}\boldsymbol{J}_{H\text{-}H}$ = 8 Hz, naphthalene, 8 H), 6.65 (d, $^{3}\boldsymbol{J}_{H\text{-}H}$ = 7 Hz, naphthalene., 8 H) ppm. Anal. Calc. for $C_{72}H_{48}B_4N_4O_8$ (1140.436): C, 75.83; H, 4.24; N, 4.91; Found: C, 75.64, H, 4.09, 4.68.

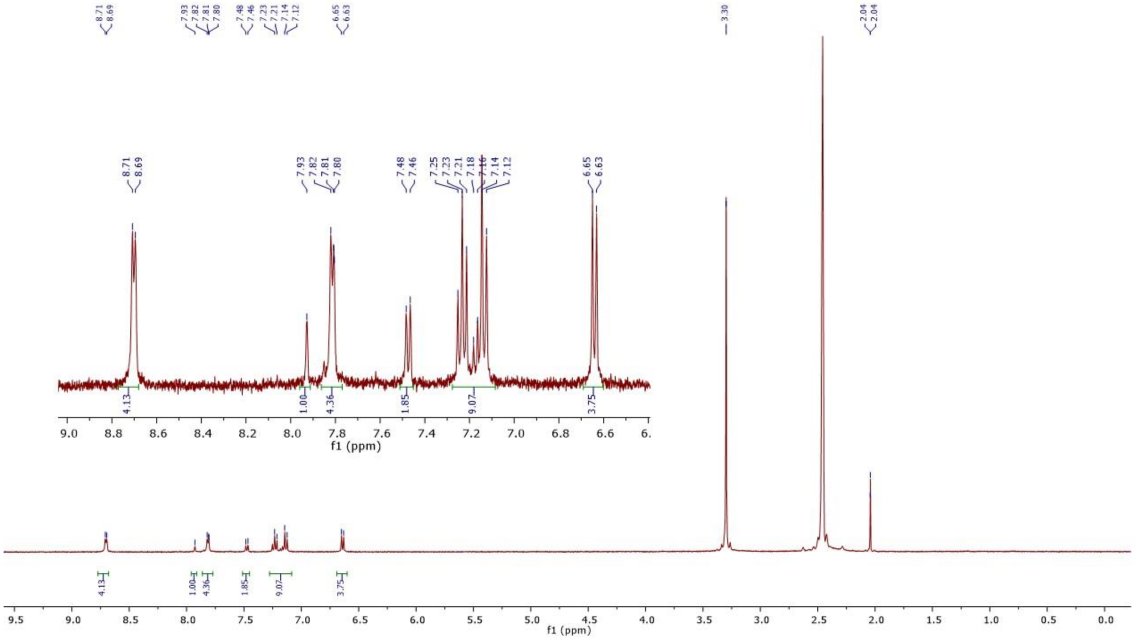

Figure S18. ^{1}H NMR spectrum of **4** in DMSO-d_6.

2.9. General procedure for the reaction of 3,4,5-trifluorophenylboronic acid with 1,8-dihydroxynaphthalene and 4,4'-bipyridine in various aromatic solvents

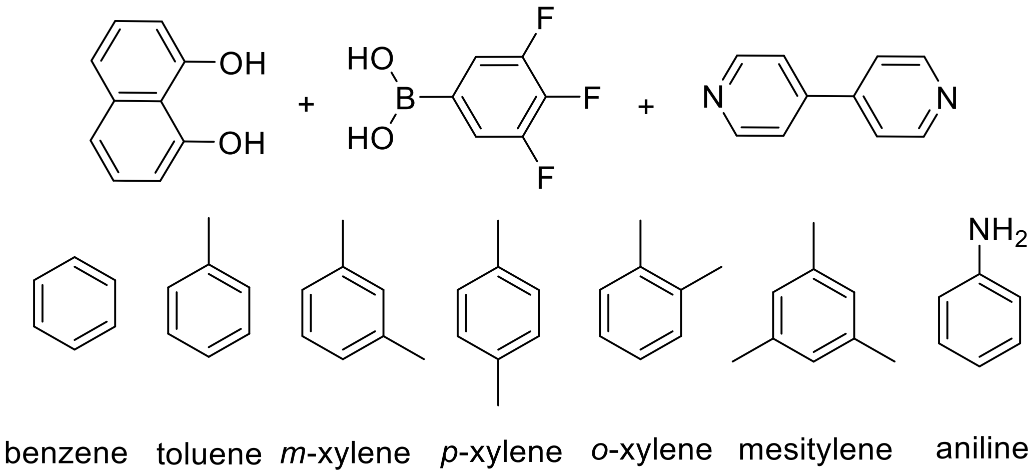

A 4 mL scintillation vial was charged with 3,4,5-trifluorophenylboronic acid (22 mg, 0.12 mmol), 1,8-dihydroxynaphthalene (20 mg, 0.12 mmol), 4,4'-bipyridine (10 mg, 0.06 mmol) and ca. 2 mL of the respective aromatic solvent or solvent mixture. Then, the mixture was heated until all the starting material had dissolved. Upon cooling to room temperature crystals formed, which were collected by filtration over a glass frit. The crystals were washed with the respective solvent, with hexanes, and dried in air for ca. 24 hours.

2.9.1 **2d**×benzene

Yield 29 mg (55 %). M.p. 209-211 °C. ^{1}H NMR (400 MHz, D1 = 40 sec., acetone-d_6): δ = 9.23 (d, $^{3}J_{H-H}$ = 4 Hz, CH-bipyridine, 4 H), 8.22 (d, $^{2}\boldsymbol{J}_{H-H}$ = 4 Hz, CH-bipyridine, 4 H), 7.58 (t, $^{3}\boldsymbol{J}_{H-F}$ = 8 Hz, $C_6F_3H_2$, 4 H), 7.46 (s, CH-benzene, 8.3 H) 7.38-7.42 (m, CH-naphthalene, 4 H), 7.00 (d, $^{3}\boldsymbol{J}_{H-H}$ = 8 Hz, CH-naphthalene, 4 H) ppm. Anal. Calc. for $C_{42}H_{24}B_2F_6N_2O_4$×$(C_6H_6)_{1.5}$ (873.445): C, 70.13; H, 3.81; N, 3.21; Found: C, 68.74, H, 3.59, 3.28.

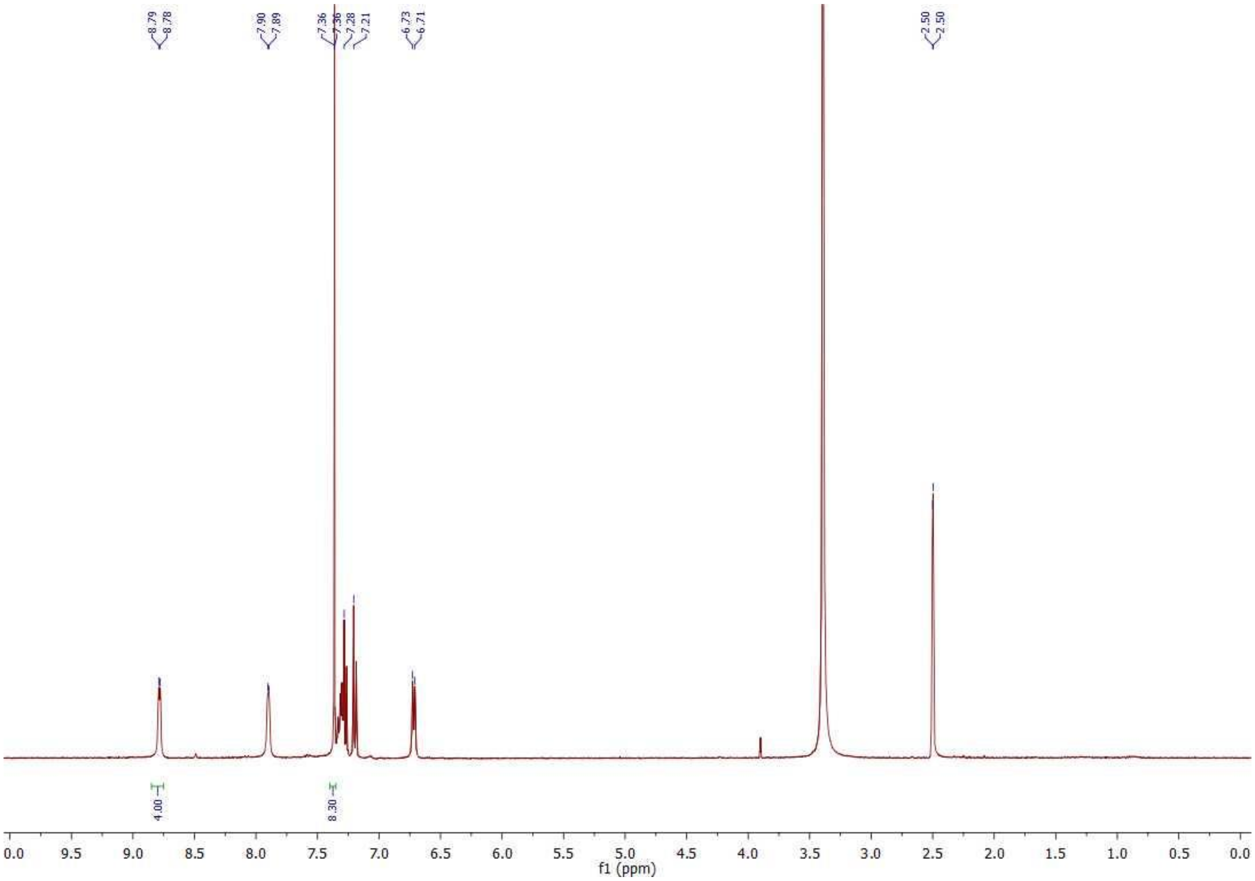

Figure S19. ^{1}H NMR spectrum of **2d**×benzene in acetone-d_6.

<u>2.9.2 **2d**×toluene</u>

Yield 29 mg (57%). M.p. 209-211 °C. ^{1}H NMR (400 MHz, DMSO-d_6): δ = 8.85 (d, $^3\boldsymbol{J}_{H\text{-}H}$ = 4 Hz, CH-bipyridine, 4 H), 7.96 (d, $^3\boldsymbol{J}_{H\text{-}H}$ = 4 Hz, CH-bipyridine, 4 H), 7.16-7.39 (m, CH-naphthalene/toluene/$C_6F_3H_2$, 17 H), 6.78 (d, $^3\boldsymbol{J}_{H\text{-}H}$ = 8 Hz, CH-naphthalene, 4 H), 2.34 (s, CH_3, 3 H) ppm. Anal. Calc. for $C_{42}H_{24}B_2F_6N_2O_4$×C_7H_8 (848.415): C, 69.37; H, 3.80; N, 3.30, Found: C, 68.77, H, 3.48, N, 3.34.

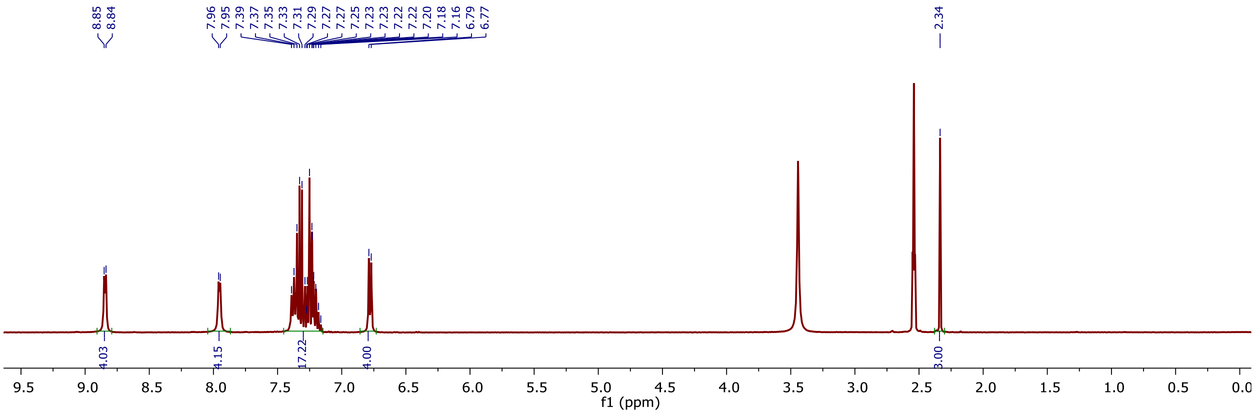

Figure S20. ^{1}H NMR spectrum of **2d**×toluene in DMSO-d6.

<u>2.9.3 **2d**×*m*-xylene</u>

Yield 37 mg (71%). M.p. 210-212 °C. ^{1}H NMR (400 MHz, DMSO-d6): δ = 8.88 (d, $^{3}\boldsymbol{J}_{H\text{-}H}$ = 4 Hz, CH-bipyridine, 4 H), 7.98 (d, $^{3}\boldsymbol{J}_{H\text{-}H}$ = 4 Hz, CH-bipyridine, 4 H), 7.24-7.42 (m, CH-naphthalene/$C_6F_3H_2$, 12 H), 7.15-7.18 (m, CH-*m*-xylene, 1.38 H), 7.00-7.02 (m, CH-*m*-xylene, 3.54 H), 6.80-6.81 (d, $^{3}\boldsymbol{J}_{H\text{-}H}$ = 4 Hz, CH-naphthalene, 4 H), 2.29 (s, CH_3, 6.4 H) ppm. Anal. Calc. for $C_{42}H_{24}B_2F_6N_2O_4$×C_8H_{10} (862.442): C, 69.63; H, 3.97, N, 3.25; Found: C, 69.22, H, 3.42, N, 3.26.

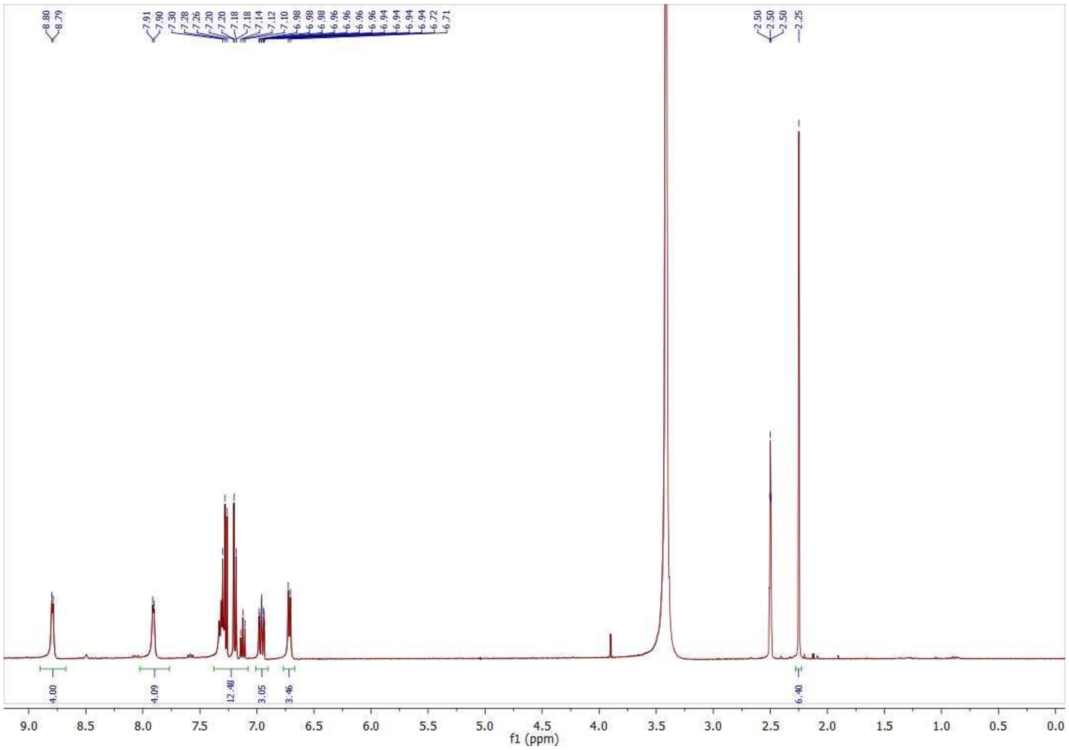

Figure S21. ^{1}H NMR spectrum of **2d**×*m*-xylene in DMSO-d6.

2.9.4 **2d**×*p*-xylene

Yield 42 mg (81%). M.p. 210-212°C. ^{1}H NMR (400 MHz, DMSO-d$_6$): δ = 8.85 (d, ^{3}***J***$_{H-H}$ = 4 Hz, CH-bipyridine, 4 H), 7.95 (d, ^{3}***J***$_{H-H}$ = 4 Hz, CH-bipyridine, 4 H), 7.31-7.39 (m, CH, 12 H), 7.09 (s, CH-arom., 4 H), 6.78 (d, ^{3}***J***$_{H-H}$ = 8 Hz, CH-arom., 4 H), 2.28 (s, CH$_3$, 6.3 H) ppm. Anal. Calc. for $C_{42}H_{24}B_2F_6N_2O_4$×C_8H_{10} (862.442): C, 69.63; H, 3.97; N, 3.25; Found: C, 68.84, H, 3.68, N, 3.29.

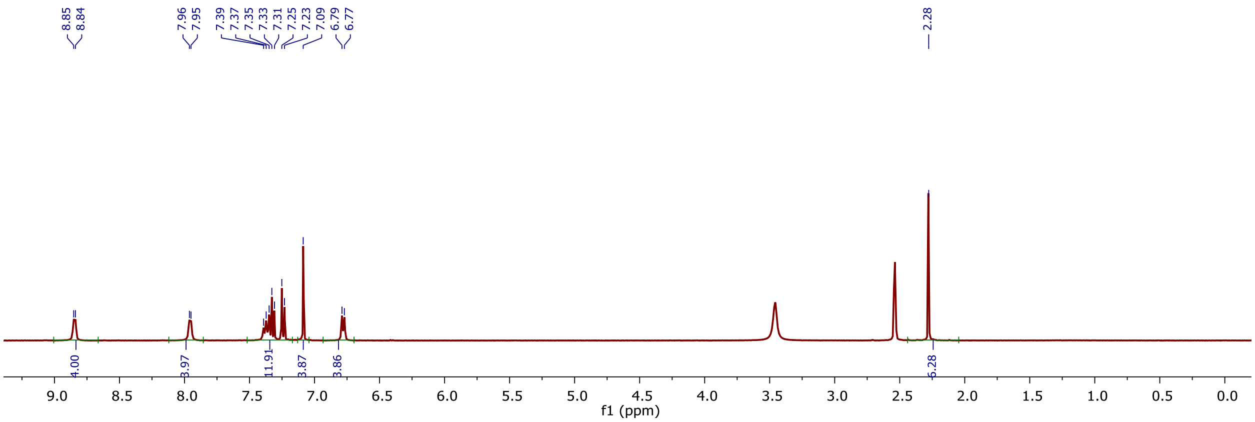

Figure S22. ^{1}H NMR spectrum of **2d**×*p*-xylene in DMSO-d$_6$.

2.9.5 **2d**×*o*-xylene

Yield 40 mg (77%). M.p. 211-212 °C. ^{1}H NMR (400 MHz, DMSO-d$_6$): δ = 8.86 (d, ^{3}***J***$_{H-H}$ = 8 Hz, CH-bipyridine, 4 H), 7.97 (d, ^{3}***J***$_{H-H}$ = 4 Hz, CH-bipyridine, 4 H), 7.31-7.40 (m, CH-naphthalene/$C_6F_3H_2$, 8 H), 7.24 (d, ^{3}***J***$_{H-H}$ = 8 Hz, CH-naphthalene, 4 H), 7.08-7.16 (m, CH-*o*-xylene, 4 H) 6.79 (d, ^{3}***J***$_{H-H}$ = 8Hz, CH-naphthalene, 4 H), 2.24 (s, CH$_3$, 6.3 H) ppm. Anal. Calc. for $C_{42}H_{24}B_2F_6N_2O_4$×C_8H_{10} (862.442): C, 69.63; H, 3.97; N, 3.25, Found: C, 69.50, H, 3.86, N, 3.28.

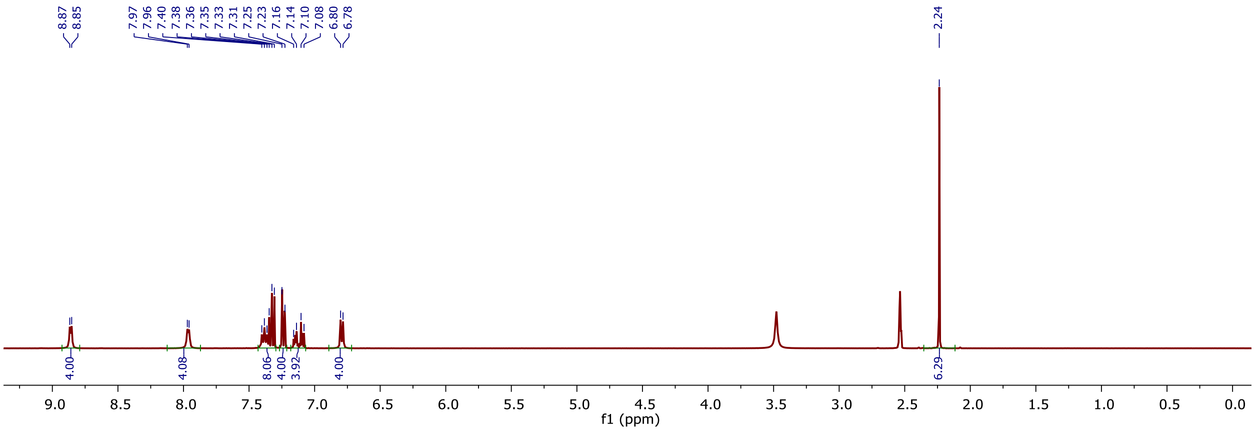

Figure S23. ^{1}H NMR spectrum of **2d**×*o*-xylene in DMSO-d$_6$.

2.9.6 **2d**×mesitylene

Yield 46 mg (73%). M.p. 222-224 °C. ^{1}H NMR (400 MHz, DMSO-d$_6$): δ = 8.84 (d, ^{3}***J***$_{H\text{-}H}$ = 8 Hz, CH-bipyridine, 4 H), 7.95 (d, ^{3}***J***$_{H\text{-}H}$ = 8 Hz, CH-bipyridine, 4 H), 7.23-7.39 (m, CH-arom., 12 H), 6.77-6.80 (m, CH-arom., 11 H), 2.25 (s, CH$_3$, 24 H) ppm. Anal. Calc. for $C_{42}H_{24}B_2F_6N_2O_4$×$(C_9H_{12})_{2.67}$ (1050.756): C, 73.62; H, 5.24; N, 2.60; Found: C, 72.43, H, 4.76, N, 2.61.

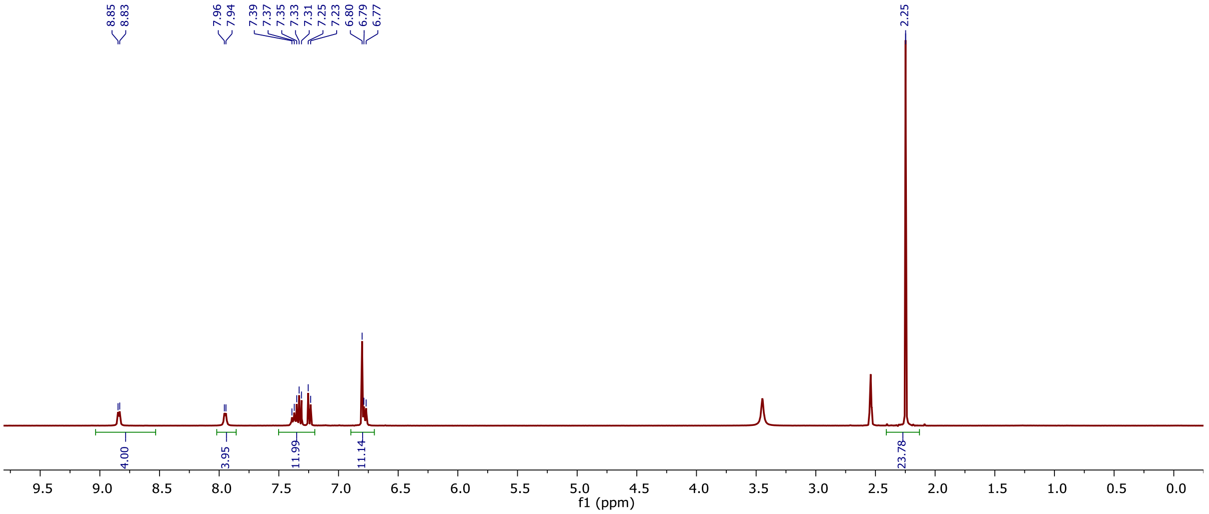

Figure S24. ^{1}H NMR spectrum of **2d**×mesitylene in DMSO-d$_6$.

2.9.7 **2d**×aniline

Yield 18 mg (33%). M.p. 197-199°C. ^{1}H NMR (400 MHz, DMSO-d_6): δ = 8.80 (d, $^3J_{H\text{-}H}$ = 8 Hz, CH-bipyridine, 4 H), 7.90 (d, $^3J_{H\text{-}H}$ = 8 Hz, CH-bipyridine, 4 H), 7.18-7.34 (m, CH-arom., 12 H), 7.03 (t, CH-arom., $^3J_{H\text{-}H}$ = 7.6 Hz, 3 H), 6.53-6.73 (m, CH-arom., 8 H), ppm. Anal. Calc. for $C_{42}H_{24}B_2F_6N_2O_4$×$(C_6H_5NH_2)_{1.5}$ (895.968): C, 68.37; H, 3.88; N, 5.47; Found: C, 68.09, H, 3.66, N, 5.29.

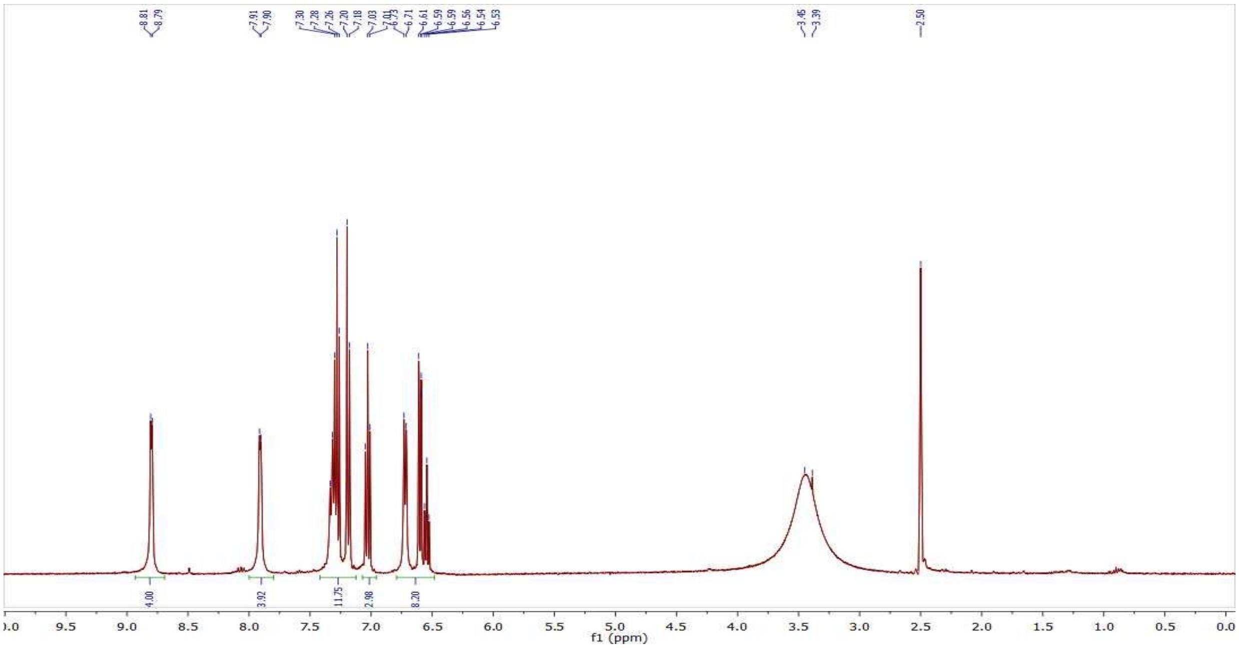

Figure S25. ^{1}H NMR spectrum of **2d**×aniline in DMSO-d_6.

2.10 Reaction of **2d** with a *o*-, *m*-, *p*-xylene mixture

After a 4 mL scintillation vial was charged with 3,4,5-trifluorophenylboronic acid (30 mg, 0.17 mmol), 1,8-dihydroxy naphthalene (29 mg, 0.18 mg), and 4,4'-bipyridine (14 mg, 0.09 mmol), 3 mL of a solution of *o*-xylene/*m*-xylene/*p*-xylene (1:1:1) was added. The mixture was gently heated until all reactants dissolved and subsequently allowed to cool to room temperature. After one day orange crystals were collected, washed several times with hexanes (3 × 1 mL), dried in air for ca. 24 hours, and analyzed by ^{1}H NMR spectroscopy.

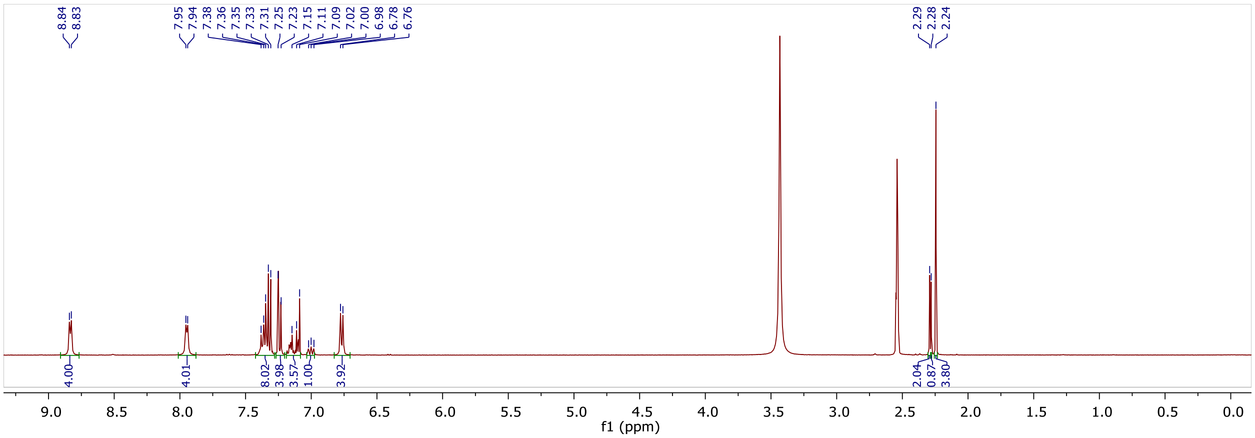

Figure S26. ^{1}H NMR spectrum (DMSO-d_6) of adduct **2d**×xylene isolated from a 1:1:1 mixture of *m*-, *p*-, and *o*-xylene.

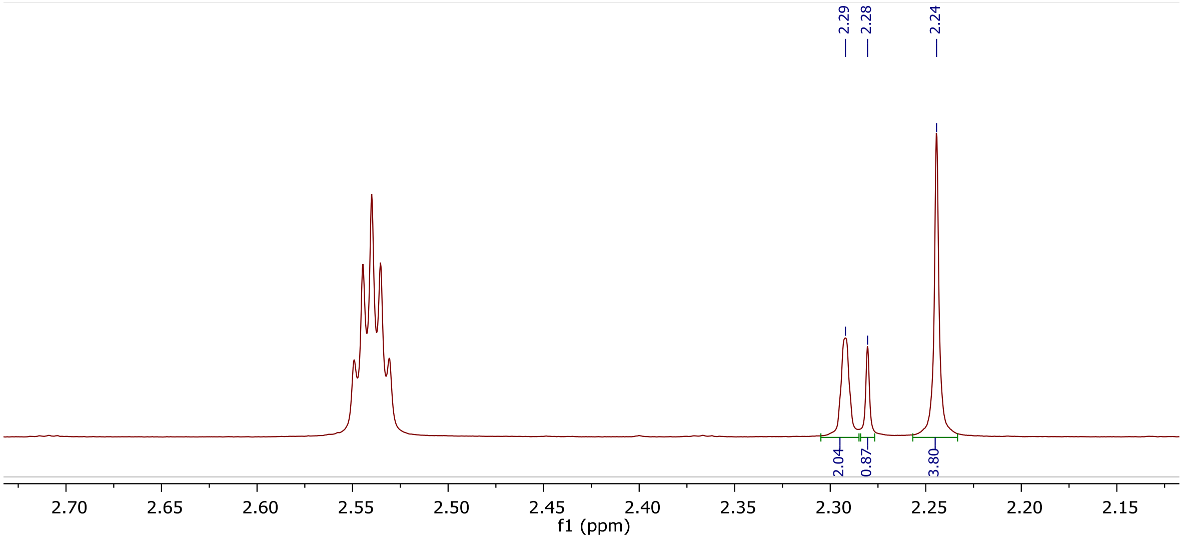

Figure S27. Aliphatic region of the ^{1}H NMR spectrum (DMSO-d_6) of adduct **2d**×xylene isolated from a 1:1:1 mixture of *m*-, *p*-, and *o*-xylene.

2.11. Procedure for soaking **2d**×*p*-xylene and **2d**×*m*-xylene in *o*-xylene

Single crystals of **2d**×*p*-xylene (ca. 15 mg) and **2d**×*m*-xylene (ca. 15 mg) were soaked in *o*-xylene (1 mL) for 24 hours. Then the crystals were collected, washed three times with hexanes, dried in vacuo, and analyzed by ^{1}H NMR spectroscopy (see below).

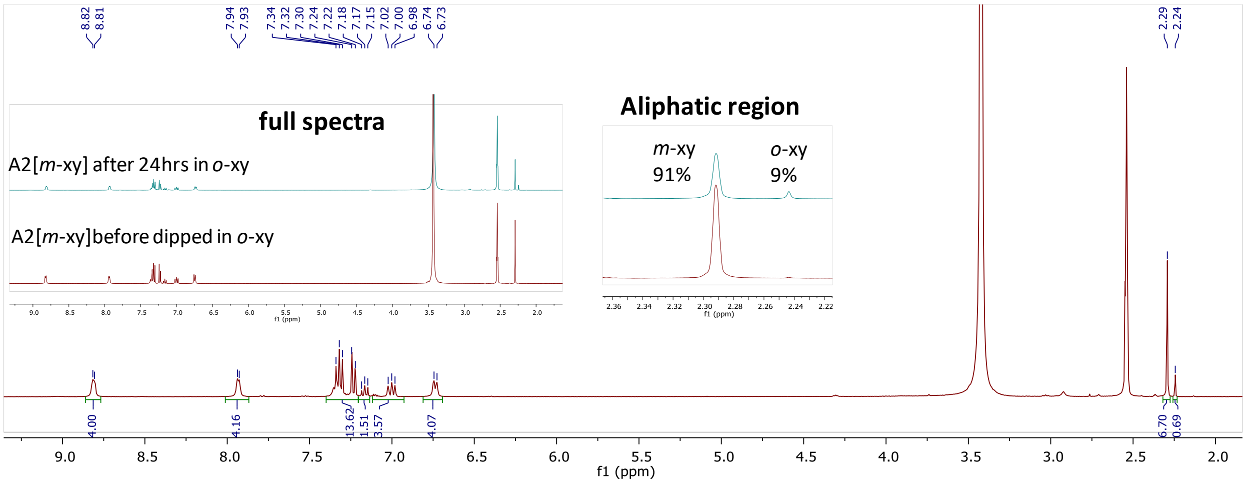

Figure S28. ^{1}H NMR spectrum (DMSO-d$_6$) of **2d**×*m*-xylene after soaking in *o*-xylene for 24 hours.

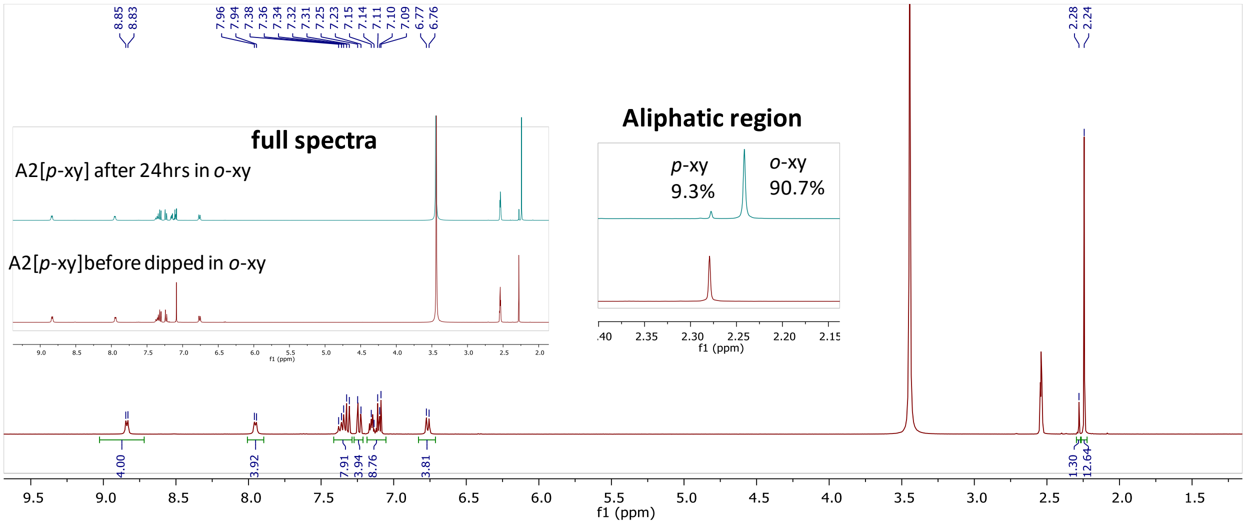

Figure S29. ^{1}H NMR spectrum (DMSO-d$_6$) of **2d**×*p*-xylene after soaking in *o*-xylene for 24 hours.

Table S1. Selected information from the thermogravimetric analysis for **2d**×solvent.

**2d**×solvent	**2d**/ solvent ratio	B. p. solvent [°C]	Peak temp. [°C]	weight loss [%]	Thermal process
**2d**×benzene	1:1.5	80	131	13.0	solvent evaporation
**2d**×toluene	1:1	111	147	11.0	solvent evaporation
**2d**×*o*-xylene	1:1	144	133	14.1	solvent evaporation
**2d**×*m*-xylene	1:1	139	122	13.5	solvent evaporation
**2d**×*p*-xylene	1:1	138	136	13.1	solvent evaporation
**2d**×mesitylene	1:2.67	165	141	28.1	solvent evaporation
**2d**×aniline	1:1.5	184	147	16.7	solvent evaporation

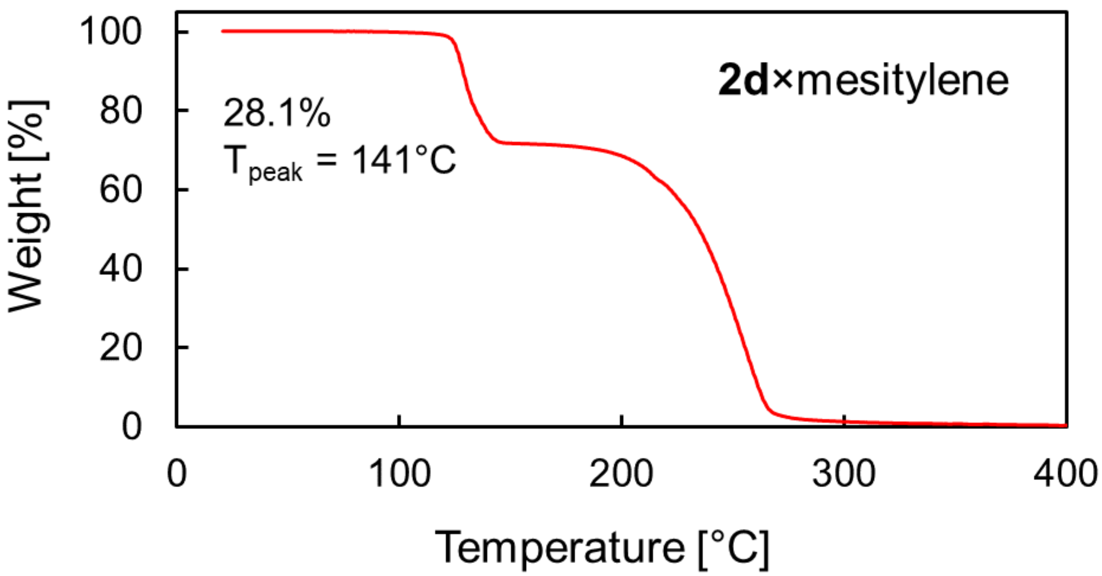

Figure S30. Thermogravimetric analysis of **2d**×mesitylene with weight loss [%] and peak temperature T_{peak} [°C] for the loss of solvent.

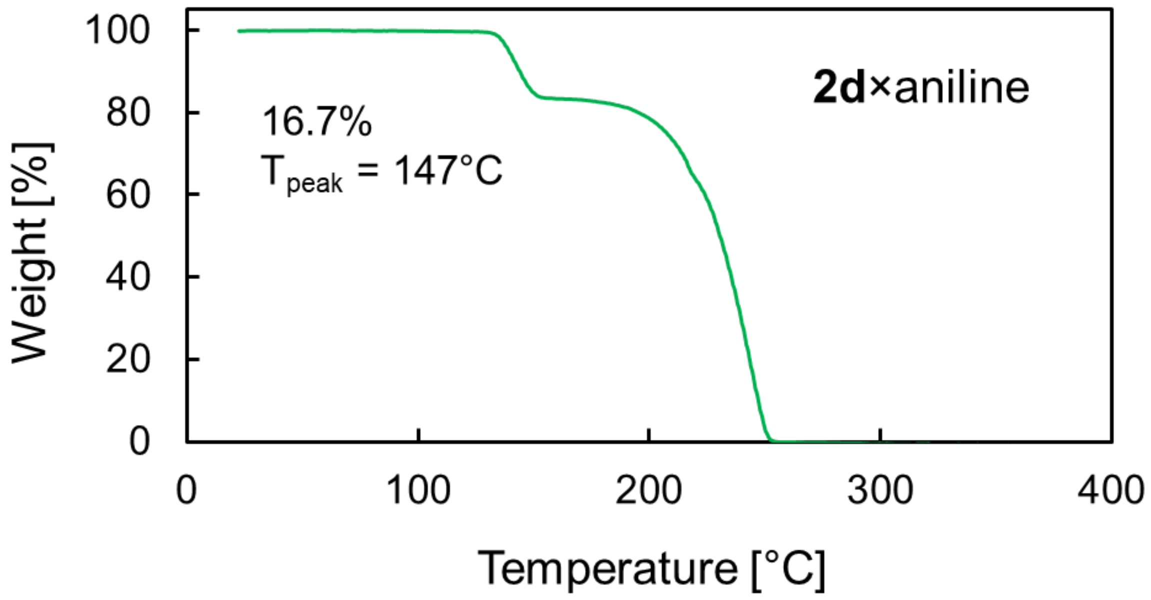

Figure S31. Thermogravimetric analysis of **2d**×aniline with weight loss [%] and peak temperature T_{peak} [°C] for the loss of solvent.

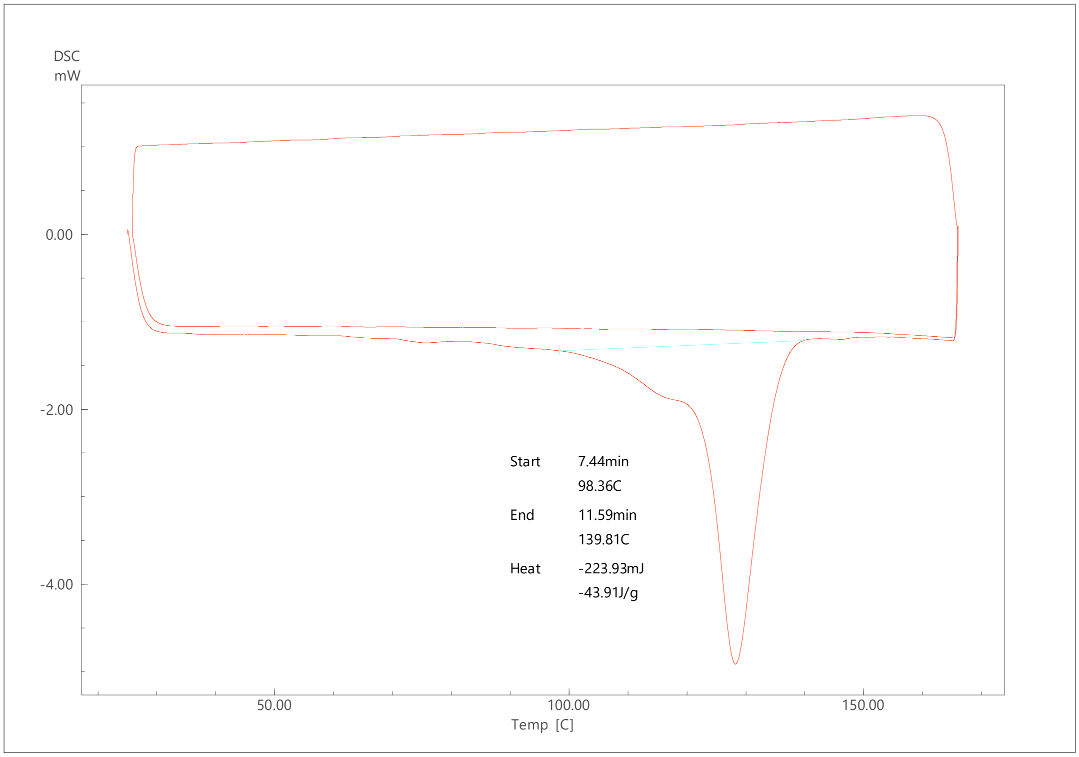

Figure S32. DSC analysis of **2d**×benzene for the loss of solvent.

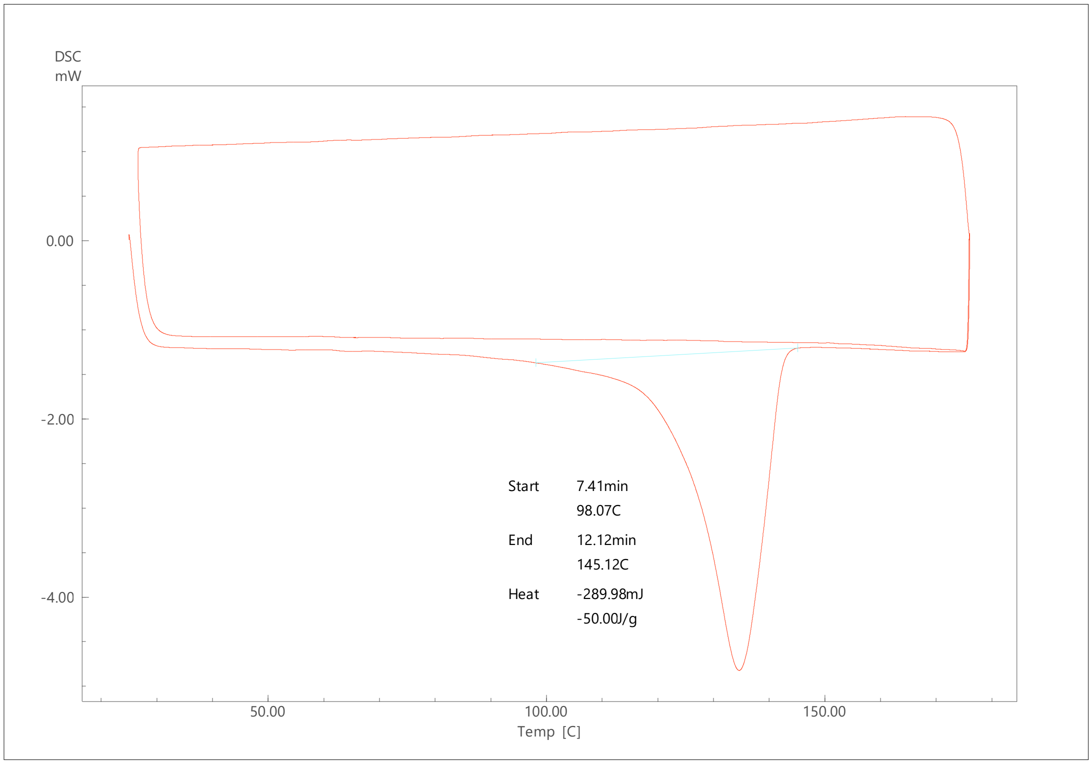

Figure S33. DSC analysis of **2d**×toluene for the loss of solvent.

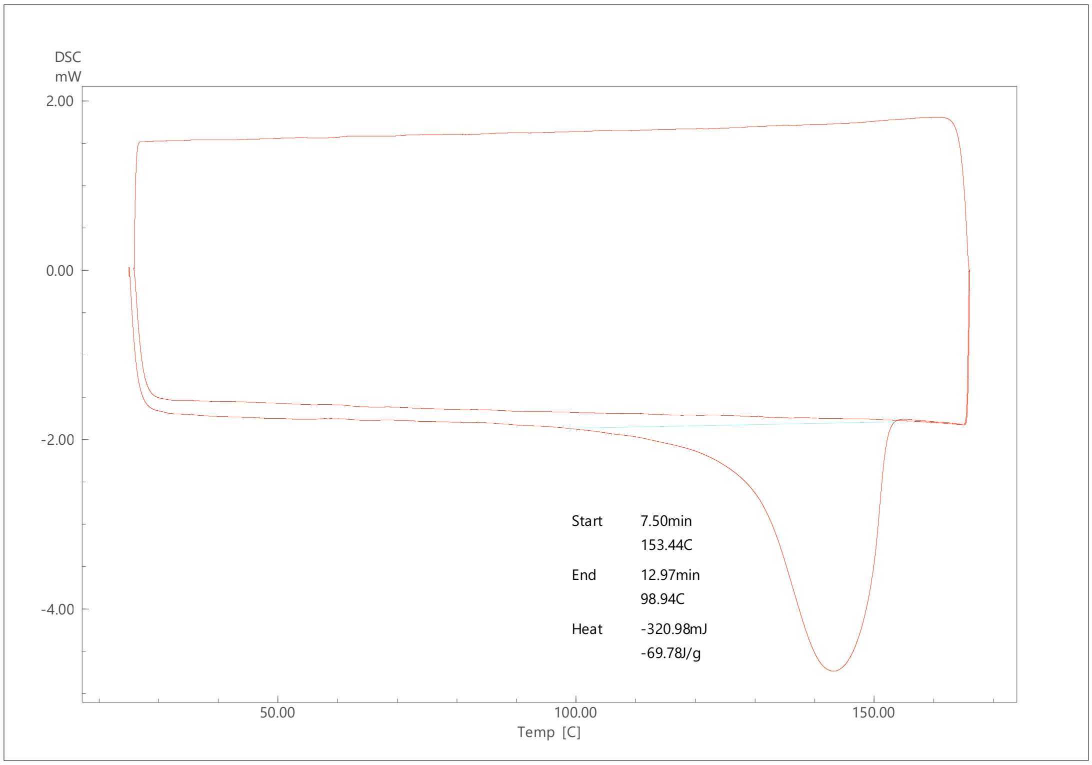

Figure S34. DSC analysis of **2d**×p-xylene for the loss of solvent.

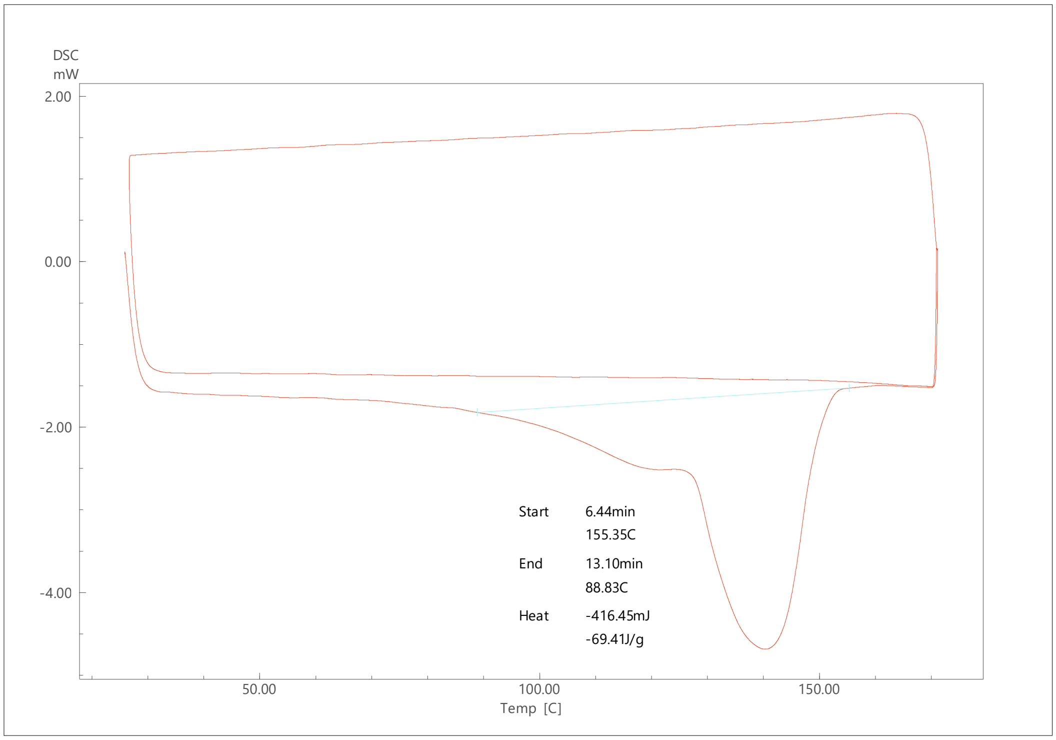

Figure S35. DSC analysis of **2d**×o-xylene for the loss of solvent.

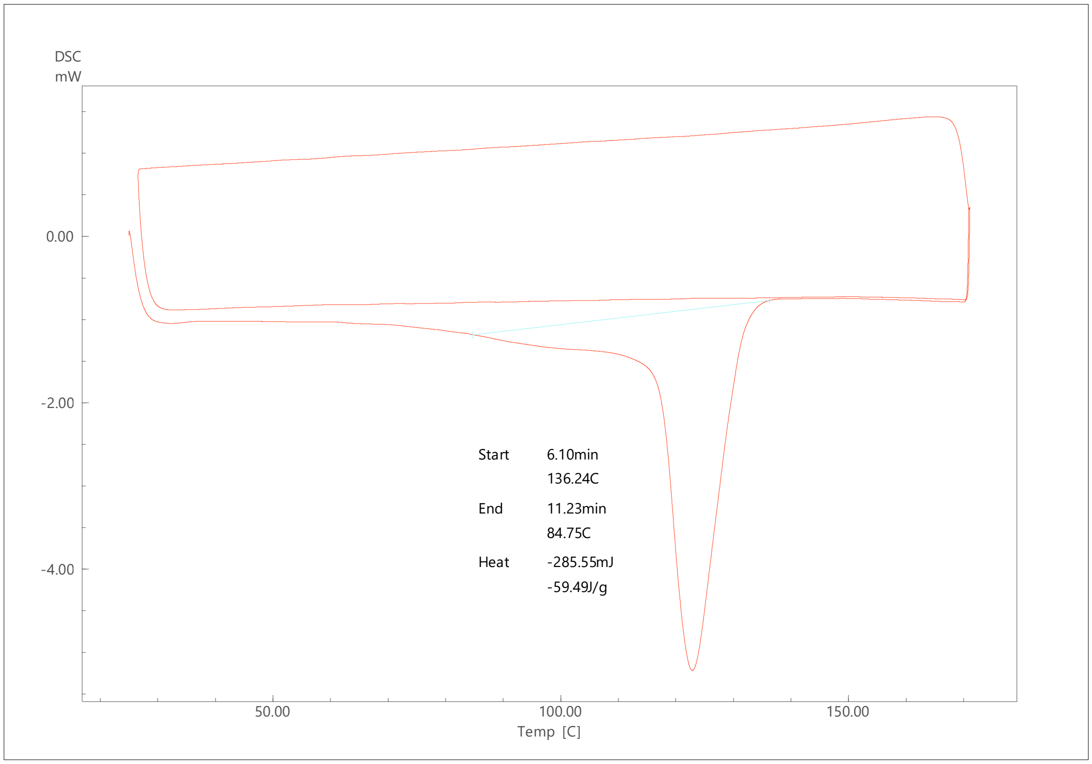

Figure S36. DSC analysis of **2d**×m-xylene for the loss of solvent.

3. X-ray Crystallography

CCDC-2252322-2252337, contain the supplementary crystallographic data for this paper. These data can be obtained from the Cambridge Crystallographic Data Centre via http://www.ccdc.cam.ac.uk/data_request/cif, or from the Cambridge Crystallographic Data Centre, 12 Union Road, Cambridge CB2 1EZ, UK; fax: (+44) 1223-336-033; e-mail: deposit@ccdc.cam.ac.uk.

3.1. **2d**×toluene - Data Collection

Data were collected on a Bruker PLATFORM three-circle diffractometer equipped with an APEX II CCD detector and operated at 1350 W (45kV, 30 mA) to generate (graphite monochromated) Mo Kα radiation (λ = 0.71073 Å). Crystals were transferred from the vial and placed on a glass slide in polyisobutylene. A Zeiss Stemi 305 microscope was used to identify a suitable specimen for X-ray diffraction from a representative sample of the material. The crystal and a small amount of the oil were collected on a MīTiGen cryoloop and transferred to the instrument where it was placed under a cold nitrogen stream (Oxford) maintained at 100K throughout the duration of the experiment. The sample was optically centered with the aid of a video camera to ensure that no translations were observed as the crystal was rotated through all positions. A unit cell collection was then carried out. After it was determined that the unit cell was not present in the CCDC database a sphere of data was collected. Omega scans were carried out with a 30 sec/frame exposure time and a rotation of 0.50° per frame.

After data collection, the unit cell was re-determined using a subset of the full data collection and it was found that the crystal was composed of a non-merohedral twin. Intensity data were corrected for Lorentz, polarization, and background effects using the Bruker program APEX 3. A semi-empirical correction for adsorption was applied using the program *SADABS*[1]. The *SHELXL-2014*[2], series of programs was used for the solution and refinement of the crystal structure. Hydrogen atoms bound to carbon and nitrogen atoms were located in the difference Fourier map and were geometrically constrained using the appropriate AFIX commands.

1. Bruker (2021) SADABS v2016/2. Bruker AXS Inc., Madison, Wisconsin, USA.
2. Sheldrick, G. M. (2015) *Acta Crystallogr.*, C**71**, 3-8.

Table S2. Crystal data and structure refinement for **2d**×toluene.

Identification code	kre18_12
Crystal Color	yellow
Crystal Habit	dendritic
Empirical formula	C49 H32 B2 F6 N2 O4
Formula weight	848.38
Temperature	100(2) K
Wavelength	0.71073 A
Crystal system	Monoclinic
Space group	$P2_1/n$
Unit cell dimensions	a = 13.635(6) Å alpha = 90 °.
	b = 15.108(6) Å beta = 92.940(5) °.
	c = 20.541(8) Å gamma = 90 °.
Volume	4226(3) Å^3
Z	4
Calculated density	1.334 Mg/m^3
Absorption coefficient	0.102 mm^{-1}
F(000)	1744
Crystal size	0.485 x 0.360 x 0.030 mm
Theta range for data collection	1.674 to 25.394 °.
Limiting indices	-16<=h<=16, -18<=k<=18, -24<=l<=24
Reflections collected / unique	7752 / 7752 [R(int) = 0.0449]
Completeness to theta = 25.242°	100.0 %
Refinement method	Full-matrix least-squares on F^2
Data / restraints / parameters	7752 / 0 / 569
Goodness-of-fit on F^2	0.982
Final R indices [I>2sigma(I)]	R1 = 0.0514, wR2 = 0.1102
R indices (all data)	R1 = 0.1050, wR2 = 0.1262
Largest diff. peak and hole	0.200 and -0.258 e.A^{-3}

3.2. **6** - Data Collection

Data were collected on a Bruker PLATFORM three circle diffractometer equipped with an APEX II CCD detector and operated at 1350 W (45kV, 30 mA) to generate (graphite monochromated) Mo Kα radiation (λ = 0.71073 Å). Crystals were transferred from the vial and placed on a glass slide in polyisobutylene. A Zeiss Stemi 305 microscope was used to identify a suitable specimen for X-ray diffraction from a representative sample of the material. The crystal and a small amount of the oil were collected on a MīTiGen cryoloop and transferred to the instrument where it was placed under a cold nitrogen stream (Oxford) maintained at 100K throughout the duration of the experiment. The sample was optically centered with the aid of a video camera to ensure that no translations were observed as the crystal was rotated through all positions.

A unit cell collection was then carried out. After it was determined that the unit cell was not present in the CCDC database a sphere of data was collected. Omega scans were carried out with a 5 sec/frame exposure time and a rotation of 0.50° per frame.

After data collection, the unit cell was re-determined using a subset of the full data collection. Intensity data were corrected for Lorentz, polarization, and background effects using the Bruker program APEX 3. A semi-empirical correction for adsorption was applied using the program *SADABS*[1]. The *SHELXL-2014*[2], series of programs was used for the solution and refinement of the crystal structure. Hydrogen atoms bound to carbon atoms were located in the difference Fourier map and were geometrically constrained using the appropriate AFIX commands.

1. Bruker (2021) SADABS v2016/2. Bruker AXS Inc., Madison, Wisconsin, USA.
2. Sheldrick, G. M. (2015) *Acta Crystallogr.*, C**71**, 3-8.

Table S3. Crystal data and structure refinement for **6**.

Identification code	kre18_09
Crystal Color	yellow
Crystal Habit	block
Empirical formula	C50 H46 B2 N2 O4
Formula weight	760.51
Temperature	100(2) K
Wavelength	0.71073 A
Crystal system	Monoclinic
Space group	$P2_1/n$
Unit cell dimensions	a = 9.8897(9) Å alpha = 90 °.
	b = 14.9121(14) Å beta = 95.6920(10) °.
	c = 13.5755(12) Å gamma = 90 °.
Volume	1992.2(3) Å^3
Z	2
Calculated density	1.268 Mg/m^3
Absorption coefficient	0.079 mm^{-1}
F(000)	804
Crystal size	0.480 x 0.405 x 0.375 mm
Theta range for data collection	2.034 to 27.226 °.
Limiting indices	-12<=h<=12, -19<=k<=19, -17<=l<=17
Reflections collected / unique	23024 / 4445 [R(int) = 0.0329]
Completeness to theta = 25.242°	100.0 %
Refinement method	Full-matrix least-squares on F^2
Data / restraints / parameters	4445 / 0 / 265
Goodness-of-fit on F^2	1.058
Final R indices [I>2sigma(I)]	R1 = 0.0381, wR2 = 0.0900
R indices (all data)	R1 = 0.0476, wR2 = 0.0954
Largest diff. peak and hole	0.335 and -0.183 e.A^{-3}

3.3. **2b** - Data Collection

Data were collected on a Bruker PLATFORM three circle diffractometer equipped with an APEX II CCD detector and operated at 1500 W (50kV, 30 mA) to generate (graphite monochromated) Mo Kα radiation (λ = 0.71073 Å). Crystals were transferred from the vial and placed on a glass slide in polyisobutylene. A Zeiss Stemi 305 microscope was used to identify a suitable specimen for X-ray diffraction from a representative sample of the material. The crystal and a small amount of the oil were collected on a MīTiGen cryoloop and transferred to the instrument where it was placed under a cold nitrogen stream (Oxford) maintained at 100K throughout the duration of the experiment. The sample was optically centered with the aid of a video camera to ensure that no translations were observed as the crystal was rotated through all positions.

A unit cell collection was then carried out. After it was determined that the unit cell was not present in the CCDC database a sphere of data was collected. Omega scans were carried out with a 40 sec/frame exposure time and a rotation of 0.50° per frame.

After data collection, the unit cell was re-determined using a subset of the full data collection. Intensity data were corrected for Lorentz, polarization, and background effects using the Bruker program APEX 3. A semi-empirical correction for adsorption was applied using the program *SADABS*[1]. The *SHELXL-2014*[2], series of programs was used for the solution and refinement of the crystal structure. Hydrogen atoms bound to carbon atoms were located in the difference Fourier map and were geometrically constrained using the appropriate AFIX commands. During the final refinement the RIGU restraint was used globally.

1. Bruker (2021) SADABS v2016/2. Bruker AXS Inc., Madison, Wisconsin, USA.
2. Sheldrick, G. M. (2015) *Acta Crystallogr.*, C71, 3-8.

Table S4. Crystal data and structure refinement for **2b**.

Identification code	kre17_30
Crystal Color	light red
Crystal Habit	block
Empirical formula	C45 H32 B2 Cl4 N2 O5
Formula weight	844.14
Temperature	100(2) K
Wavelength	0.71073 A
Crystal system	Monoclinic
Space group	$P2_1/n$
Unit cell dimensions	a = 15.0088(15) Å alpha = 90 °.
	b = 14.6683(14) Å beta = 109.5970(10) °.
	c = 18.9539(19) Å gamma = 90 °.
Volume	3931.1(7) Å^3
Z	4
Calculated density	1.426 Mg/m^3
Absorption coefficient	0.353 mm^{-1}
F(000)	1736
Crystal size	0.450 x 0.230 x 0.120 mm
Theta range for data collection	1.508 to 27.195 °.
Limiting indices	-19<=h<=19, -18<=k<=18, -24<=l<=24
Reflections collected / unique	45743 / 8733 [R(int) = 0.0300]
Completeness to theta = 25.242°	100.0 %
Refinement method	Full-matrix least-squares on F^2
Data / restraints / parameters	8733 / 495 / 525
Goodness-of-fit on F^2	1.028
Final R indices [I>2sigma(I)]	R1 = 0.0353, wR2 = 0.0843
R indices (all data)	R1 = 0.0482, wR2 = 0.0918
Largest diff. peak and hole	0.320 and -0.456 e.A^{-3}

3.4. **2d**×benzene - Data Collection

Data were collected on a Bruker PLATFORM three circle diffractometer equipped with an APEX II CCD detector and operated at 1350 W (45kV, 30 mA) to generate (graphite monochromated) Mo Kα radiation (λ = 0.71073 Å). Crystals were transferred from the vial and placed on a glass slide in polyisobutylene. A Zeiss Stemi 305 microscope was used to identify a suitable specimen for X-ray diffraction from a representative sample of the material. The crystal and a small amount of the oil were collected on a MīTiGen cryoloop and transferred to the instrument where it was placed under a cold nitrogen stream (Oxford) maintained at 100K throughout the duration of the experiment. The sample was optically centered with the aid of a video camera to ensure that no translations were observed as the crystal was rotated through all positions.

A unit cell collection was then carried out. After it was determined that the unit cell was not present in the CCDC database a sphere of data was collected. Omega scans were carried out with a 20 sec/frame exposure time and a rotation of 0.50° per frame.

After data collection, the unit cell was re-determined using a subset of the full data collection. Intensity data were corrected for Lorentz, polarization, and background effects using the Bruker program APEX 3. A semi-empirical correction for adsorption was applied using the program *SADABS*[1]. The *SHELXL-2014*[2], series of programs was used for the solution and refinement of the crystal structure. Within the structure there was a significant amount of positional disorder that was modeled as split sites. For carbon atoms C85 < C96 the sites were split into parts A and B, and allowed to free refine their site occupancies to a total value of 1. The site occupancy values were 0.47 and 0.53 for part A and B, respectively. For carbon atoms C69 < C74 and fluorine atoms F10 < F12 the sites were also split into parts A and B with site occupancies of 0.65 and 0.35, respectively. The rigid-bond restraint RIGU was also applied globally with the AFIX 66 constraint used on the disordered interstitial benzene rings. Hydrogen atoms bound to carbon atoms were located in the difference Fourier map and were geometrically constrained using the appropriate AFIX commands. Also, with the structure there were two crystallographically unique molecules, resulting in a Z’ value of 2 for the structure.

1. Bruker (2021) SADABS v2016/2. Bruker AXS Inc., Madison, Wisconsin, USA.
2. Sheldrick, G. M. (2015) *Acta Crystallogr.*, C**71**, 3-8.

Table S5. Crystal data and structure refinement for **2d**×benzene.

Identification code	kre18_16
Crystal Color	yellow
Crystal Habit	chunk
Empirical formula	C102 H66 B4 F12 N4 O8
Formula weight	1746.82
Temperature	173(2) K
Wavelength	0.71073 A
Crystal system	Triclinic
Space group	$P\bar{1}$
Unit cell dimensions	a = 14.0559(19) Å alpha = 98.286(2) °.
	b = 14.5069(19) Å beta = 91.825(2) °.
	c = 20.930(3) Å gamma = 94.442(2) °.
Volume	4206.6(9) $Å^3$
Z	2
Calculated density	1.379 Mg/m^3
Absorption coefficient	0.104 mm^{-1}
F(000)	1796
Crystal size	0.450 x 0.240 x 0.180 mm
Theta range for data collection	0.984 to 25.722 °.
Limiting indices	-17<=h<=17, -17<=k<=17, -25<=l<=25
Reflections collected / unique	50128 / 15972 [R(int) = 0.0450]
Completeness to theta = 25.242°	99.9 %
Refinement method	Full-matrix least-squares on F^2
Data / restraints / parameters	15972 / 1275 / 1279
Goodness-of-fit on F^2	1.020
Final R indices [I>2sigma(I)]	R1 = 0.0487, wR2 = 0.1100
R indices (all data)	R1 = 0.0978, wR2 = 0.1317
Largest diff. peak and hole	0.207 and -0.278 $e.A^{-3}$

3.5. **2d**×*p*-xylene - Data Collection

Data were collected on a Bruker PLATFORM three circle diffractometer equipped with an APEX II CCD detector and operated at 1350 W (40kV, 30 mA) to generate (graphite monochromated) Mo Kα radiation (λ = 0.71073 Å). Crystals were transferred from the vial and placed on a glass slide in polyisobutylene. A Zeiss Stemi 305 microscope was used to identify a suitable specimen for X-ray diffraction from a representative sample of the material. The crystal and a small amount of the oil were collected on a MīTiGen cryoloop and transferred to the instrument where it was placed under a cold nitrogen stream (Oxford) maintained at 100K throughout the duration of the experiment. The sample was optically centered with the aid of a video camera to ensure that no translations were observed as the crystal was rotated through all positions.

A unit cell collection was then carried out. After it was determined that the unit cell was not present in the CCDC database a sphere of data was collected. Omega scans were carried out with a 40 sec/frame exposure time and a rotation of 0.50° per frame.

After data collection, the unit cell was re-determined using a subset of the full data collection. Intensity data were corrected for Lorentz, polarization, and background effects using the Bruker program APEX 3. A semi-empirical correction for adsorption was applied using the program *SADABS*[1]. The *SHELXL-2014*[2], series of programs was used for the solution and refinement of the crystal structure. Within the structure, there was rotational disorder of the 1,2,3-trifluorobenzene molecule containing atoms F4, F5, F6, and C27 < C32, about C31. The atom sites were split into 2 sites, A and B, and their site occupancies were allowed to free refine to a total occupancy of 1. The final site occupancies of the two positions were 0.61 and 0.39 for parts A and B, respectively. The rigid-bond restraint RIGU was also applied globally along with SIMU and DELU restraints for the disordered trifluorobenzene moiety. Hydrogen atoms bound to carbon atoms were located in the difference Fourier map and were geometrically constrained using the appropriate AFIX commands.

1. Bruker (2021) SADABS v2016/2. Bruker AXS Inc., Madison, Wisconsin, USA.
2. Sheldrick, G. M. (2015) *Acta Crystallogr.*, C**71**, 3-8.

Table S6. Crystal data and structure refinement for **2d**×*p*-xylene.

Identification code	kre18_33
Crystal Color	yellow
Crystal Habit	chunk
Empirical formula	C50 H34 B2 F6 N2 O4
Formula weight	862.41
Temperature	100(2) K
Wavelength	0.71073 A
Crystal system	Monoclinic
Space group	$P2_1/n$
Unit cell dimensions	a = 13.949(3) Å alpha = 90 °.
	b = 15.752(4) Å beta = 106.771(3) °.
	c = 19.717(4) Å gamma = 90 °.
Volume	4147.9(16) $Å^3$
Z	4
Calculated density	1.381 Mg/m^3
Absorption coefficient	0.105 mm^{-1}
F(000)	1776
Crystal size	0.480 x 0.470 x 0.125 mm
Theta range for data collection	1.594 to 25.469 °.
Limiting indices	-16<=h<=16, -19<=k<=18, -23<=l<=23
Reflections collected / unique	41795 / 7649 [R(int) = 0.0793]
Completeness to theta = 25.242°	100.0 %
Refinement method	Full-matrix least-squares on F^2
Data / restraints / parameters	7649 / 834 / 637
Goodness-of-fit on F^2	1.026
Final R indices [I>2sigma(I)]	R1 = 0.0665, wR2 = 0.1645
R indices (all data)	R1 = 0.1158, wR2 = 0.1935
Largest diff. peak and hole	0.386 and -0.394 $e.A^{-3}$

3.6. **2d**×*m*-xylene - Data Collection

Data were collected on a Bruker PLATFORM three circle diffractometer equipped with an APEX II CCD detector and operated at 1350 W (45kV, 30 mA) to generate (graphite monochromated) Mo Kα radiation (λ = 0.71073 Å). Crystals were transferred from the vial and placed on a glass slide in polyisobutylene. A Zeiss Stemi 305 microscope was used to identify a suitable specimen for X-ray diffraction from a representative sample of the material. The crystal and a small amount of the oil were collected on a MīTiGen cryoloop and transferred to the instrument where it was placed under a cold nitrogen stream (Oxford) maintained at 100K throughout the duration of the experiment. The sample was optically centered with the aid of a video camera to ensure that no translations were observed as the crystal was rotated through all positions.

A unit cell collection was then carried out. After it was determined that the unit cell was not present in the CCDC database a sphere of data was collected. Omega scans were carried out with a 20 sec/frame exposure time and a rotation of 0.50° per frame.

After data collection, the unit cell was re-determined using a subset of the full data collection. Intensity data were corrected for Lorentz, polarization, and background effects using the Bruker program APEX 3. A semi-empirical correction for adsorption was applied using the program *SADABS*[1]. The *SHELXL-2014*[2], series of programs was used for the solution and refinement of the crystal structure. Within the structure one of the meta-xylene interstitial molecules was positionally disordered. For the molecule containing carbon atoms C183 < C188 and C196 the occupancy was 0.53, while carbon atoms C189 < C194 and C197 had an occupancy of 0.47. Also within the structure there were three crystallographically unique molecules, resulting in a *Z*' value of 3 for the structure.

1. Bruker (2021) SADABS v2016/2. Bruker AXS Inc., Madison, Wisconsin, USA.
2. Sheldrick, G. M. (2015) *Acta Crystallogr.*, C**71**, 3-8.

Table S7. Crystal data and structure refinement for **2d**×*m*-xylene.

Identification code	kre18_24
Crystal Color	yellow
Crystal Habit	block
Empirical formula	C190 H152 B6 F18 N6 O12
Formula weight	3118.03
Temperature	100(2) K
Wavelength	0.71073 A
Crystal system	Triclinic
Space group	$P\bar{1}$
Unit cell dimensions	a = 20.516(3) Å alpha = 110.674(2) °.
	b = 20.566(3) Å beta = 106.679(2) °.
	c = 20.978(3) Å gamma = 91.694(2) °.
Volume	7848(2) $Å^3$
Z	2
Calculated density	1.319 Mg/m^3
Absorption coefficient	0.096 mm^{-1}
F(000)	3244
Crystal size	0.480 x 0.405 x 0.370 mm
Theta range for data collection	1.047 to 25.402 °.
Limiting indices	-24<=h<=24, -24<=k<=24, -25<=l<=25
Reflections collected / unique	117929 / 28833 [R(int) = 0.0497]
Completeness to theta = 25.242°	100.0 %
Refinement method	Full-matrix least-squares on F^2
Data / restraints / parameters	28833 / 0 / 2146
Goodness-of-fit on F^2	1.023
Final R indices [I>2sigma(I)]	R1 = 0.0510, wR2 = 0.1281
R indices (all data)	R1 = 0.0898, wR2 = 0.1545
Largest diff. peak and hole	0.588 and -0.338 $e.A^{-3}$

3.7. **2d**×aniline - Data Collection

Data were collected on a Bruker PLATFORM three circle diffractometer equipped with an APEX II CCD detector and operated at 1350 W (40kV, 30 mA) to generate (graphite monochromated) Mo Kα radiation (λ = 0.71073 Å). Crystals were transferred from the vial and placed on a glass slide in polyisobutylene. A Zeiss Stemi 305 microscope was used to identify a suitable specimen for X-ray diffraction from a representative sample of the material. The crystal and a small amount of the oil were collected on a MīTiGen cryoloop and transferred to the instrument where it was placed under a cold nitrogen stream (Oxford) maintained at 100K throughout the duration of the experiment. The sample was optically centered with the aid of a video camera to ensure that no translations were observed as the crystal was rotated through all positions.

A unit cell collection was then carried out. After it was determined that the unit cell was not present in the CCDC database a sphere of data was collected. Omega scans were carried out with a 5 sec/frame exposure time and a rotation of 0.50° per frame.

After data collection, the unit cell was re-determined using a subset of the full data collection. Intensity data were corrected for Lorentz, polarization, and background effects using the Bruker program APEX 3. A semi-empirical correction for adsorption was applied using the program *SADABS*[1]. The *SHELXL-2014*[2], series of programs was used for the solution and refinement of the crystal structure. Within the structure, a disordered aniline molecule was positionally disorder over four sites. Nitrogen atom N6 and carbon atoms C91 < C96 were split into parts A, B, C, and D with site occupancies of 0.58, 0.07, 0.17, and 0.18, respectively. While the sites were allowed to free refine their site occupancies the total was constrained to 1 with a SUMP constraint. The rigid-bond restraint RIGU was also applied globally along with SIMU and DLEU restraints for the disordered aniline molecule. Hydrogen atoms bound to carbon and nitrogen atoms were located in the difference Fourier map where possible and were geometrically constrained using the appropriate AFIX commands. Also with the structure there were two crystallographically unique molecules, resulting in a Z’ value of 2 for the structure.

1. Bruker (2021) SADABS v2016/2. Bruker AXS Inc., Madison, Wisconsin, USA.
2. Sheldrick, G. M. (2015) *Acta Crystallogr.*, C**71**, 3-8.

Table S8. Crystal data and structure refinement for **2d**×aniline.

Identification code	kre18_32
Crystal Color	red
Crystal Habit	chunk
Empirical formula	C51 H34.50 B2 F6 N3.50 O4
Formula weight	895.94
Temperature	100(2) K
Wavelength	0.71073 A
Crystal system	Triclinic
Space group	$P\bar{1}$
Unit cell dimensions	a = 14.1479(15) Å alpha = 91.1670(10) °.
	b = 14.2447(15) Å beta = 96.9410(10) °.
	c = 20.909(2) Å gamma = 94.4800(10) °.
Volume	4168.3(8) $Å^3$
Z	4
Calculated density	1.428 Mg/m^3
Absorption coefficient	0.108 mm^{-1}
F(000)	1844
Crystal size	0.480 x 0.470 x 0.430 mm
Theta range for data collection	0.981 to 27.159 °.
Limiting indices	-18<=h<=18, -18<=k<=18, -26<=l<=26
Reflections collected / unique	98287 / 18426 [R(int) = 0.0314]
Completeness to theta = 25.242°	100.0 %
Refinement method	Full-matrix least-squares on F^2
Data / restraints / parameters	18426 / 1637 / 1344
Goodness-of-fit on F^2	1.020
Final R indices [I>2sigma(I)]	R1 = 0.0404, wR2 = 0.0957
R indices (all data)	R1 = 0.0526, wR2 = 0.1033
Largest diff. peak and hole	0.369 and -0.316 $e.A^{-3}$

3.8. **5** - Data Collection

Data were collected on a Bruker PLATFORM three circle diffractometer equipped with an APEX II CCD detector and operated at 1350 W (45kV, 30 mA) to generate (graphite monochromated) Mo Kα radiation (λ = 0.71073 Å). Crystals were transferred from the vial and placed on a glass slide in polyisobutylene. A Zeiss Stemi 305 microscope was used to identify a suitable specimen for X-ray diffraction from a representative sample of the material. The crystal and a small amount of the oil were collected on a MīTiGen cryoloop and transferred to the instrument where it was placed under a cold nitrogen stream (Oxford) maintained at 100K throughout the duration of the experiment. The sample was optically centered with the aid of a video camera to ensure that no translations were observed as the crystal was rotated through all positions.

A unit cell collection was then carried out. After it was determined that the unit cell was not present in the CCDC database a sphere of data was collected. Omega and Phi scans were carried out with a 20 sec/frame exposure time and a rotation of 0.50° per frame for low angle data (INF – 1.2 Å), while high angle data (1.4 – 0.6 Å) was carried out with a 60 sec/frame exposure time and a rotation of 0.50° per frame.

After data collection, the unit cell was re-determined using a subset of the full data collection. Intensity data were corrected for Lorentz, polarization, and background effects using the Bruker program APEX 3. A semi-empirical correction for adsorption was applied using the program *SADABS*[1]. The *SHELXL-2014*[2], series of programs was used for the solution and refinement of the crystal structure. During the initial structure solution, it was obvious that various atomic positions were disordered. The atoms contained within various molecules were allowed to free refine their site occupancies with individual variables. Overall, 5 different free variable values were used throughout the structure. In certain cases, SIMU and DELU restraints were also required to maintain reasonable thermal ellipsoids for minor components of the disordered sites. After all of the disorder within the main molecules was treated, attempts were made to model the disordered interstitial acetone molecules was well. However, these attempts were unsuccessful and the interstitial electron density was treated using the programs PLATON[3]/SQUEEZE[4], which resulted in a total of two major void spaces each containing an electron count of 574, and 8 minor void spaces with 30 electrons each. Based on the electron density of

acetone, there are approximately 52 acetone molecules scattered throughout the interstitial space within the structure. However, this atomic information was not added to the CIF file in the UNIT atom count. Hydrogen atoms bound to carbon atoms were geometrically constrained using the appropriate AFIX commands and the rigid-bond restraint RIGU was applied globally. The Z value for the structure was 32, while the Z' value was 4.

1. Bruker (2021) SADABS v2016/2. Bruker AXS Inc., Madison, Wisconsin, USA.
2. Sheldrick, G. M. (2015) *Acta Crystallogr*., C**71**, 3-8.
3. Spek, A.L. (2009). Acta Cryst. D65, 148-155.
4. Spek, A.L. (2015). Acta Cryst. C71, 9-18.

Table S9. Crystal data and structure refinement for **5**.

Identification code	kre18_05
Crystal Color	dark green
Crystal Habit	chunk
Empirical formula	C44 H34 B2 N2 O4
Formula weight	676.35
Temperature	100(2) K
Wavelength	0.71073 A
Crystal system	Triclinic
Space group	*C2/c*
Unit cell dimensions	a = 44.5048(10) Å alpha = 90 °.
	b = 22.2793(5) Å beta = 93.4800(10) °.
	c = 31.4907(7) Å gamma = 90 °.
Volume	31166.6(12) $Å^3$
Z	32
Calculated density	1.153 Mg/m^3
Absorption coefficient	0.073 mm^{-1}
F(000)	11328
Crystal size	0.450 x 0.420 x 0.250 mm
Theta range for data collection	1.195 to 25.411 °.
Limiting indices	-53<=h<=53, -26<=k<=26, -38<=l<=37
Reflections collected / unique	271389 / 28603 [R(int) = 0.0805]
Completeness to theta = 25.242°	100.0 %
Refinement method	Full-matrix least-squares on F^2
Data / restraints / parameters	28603 / 2808 / 2136
Goodness-of-fit on F^2	1.020
Final R indices [I>2sigma(I)]	R1 = 0.0629, wR2 = 0.1565
R indices (all data)	R1 = 0.1085, wR2 = 0.1863
Largest diff. peak and hole	0.445 and -0.270 e.A^{-3}

3.9. **2d** - Data Collection

Data were collected on a Bruker PLATFORM three circle diffractometer equipped with an APEX II CCD detector and operated at 1350 W (45kV, 30 mA) to generate (graphite monochromated) Mo Kα radiation (λ = 0.71073 Å). Crystals were transferred from the vial and placed on a glass slide in polyisobutylene. A Zeiss Stemi 305 microscope was used to identify a suitable specimen for X-ray diffraction from a representative sample of the material. The crystal and a small amount of the oil were collected on a MīTiGen cryoloop and transferred to the instrument where it was placed under a cold nitrogen stream (Oxford) maintained at 100K throughout the duration of the experiment. The sample was optically centered with the aid of a video camera to ensure that no translations were observed as the crystal was rotated through all positions.

A unit cell collection was then carried out. After it was determined that the unit cell was not present in the CCDC database a sphere of data was collected. Omega scans were carried out with a 5 sec/frame exposure time and a rotation of 0.50° per frame.

After data collection, the unit cell was re-determined using a subset of the full data collection. Intensity data were corrected for Lorentz, polarization, and background effects using the Bruker program APEX 3. A semi-empirical correction for adsorption was applied using the program *SADABS*[1]. The *SHELXL-2014*[2], series of programs was used for the solution and refinement of the crystal structure. Hydrogen atoms bound to carbon atoms were located in the difference Fourier map and were geometrically constrained using the appropriate AFIX commands. Due to the location of the acetone molecule consisting of atoms O6, C46, C47, and C48 to a 3-fold rotation, the site occupancies of these atoms were constrained to 0.33.

1. Bruker (2021) SADABS v2016/2. Bruker AXS Inc., Madison, Wisconsin, USA.
2. Sheldrick, G. M. (2015) *Acta Crystallogr*., C**71**, 3-8.

Table S10. Crystal data and structure refinement for **2d**.

Identification code	kre18_11
Crystal Color	red
Crystal Habit	chunk
Empirical formula	C46 H32 B2 F6 N2 O5.33
Formula weight	535.42
Temperature	100(2) K
Wavelength	0.71073 A
Crystal system	Trigonal
Space group	$R\bar{3}$
Unit cell dimensions	a = 23.5956(19) Å alpha = 90 °.
	b = 23.5956(19) Å beta = 90 °.
	c = 36.665(4) Å gamma = 120 °.
Volume	17678(3) $Å^3$
Z	18
Calculated density	1.410 Mg/m^3
Absorption coefficient	0.110 mm^{-1}
F(000)	7716
Crystal size	0.480 x 0.470 x 0.300 mm
Theta range for data collection	1.141 to 27.113 °.
Limiting indices	-30<=h<=30, -30<=k<=30, -46<=l<=46
Reflections collected / unique	69536 / 8676 [R(int) = 0.0435]
Completeness to theta = 25.242°	100.0 %
Refinement method	Full-matrix least-squares on F^2
Data / restraints / parameters	8676 / 0 / 581
Goodness-of-fit on F^2	1.028
Final R indices [I>2sigma(I)]	R1 = 0.0395, wR2 = 0.0897
R indices (all data)	R1 = 0.0553, wR2 = 0.0981
Largest diff. peak and hole	0.359 and -0.244 $e.A^{-3}$

3.10. **3** - Data Collection

Data were collected on a Rigaku SCX-Mini two-circle X-ray diffractometer equipped with a Mercury 2 CCD detector was and operated at 2000 W (50kV, 40 mA) to generate (graphite monochromated) Mo Kα radiation (λ = 0.71073 Å). Crystals were transferred from the vial and placed on a glass slide in polyisobutylene. A Zeiss Stemi 305 microscope was used to identify a suitable specimen for X-ray diffraction from a representative sample of the material. The crystal and a small amount of the oil were collected on a MīTiGen cryoloop and transferred to the instrument where it was placed under a cold nitrogen stream (Oxford) maintained at 170K throughout the duration of the experiment. The sample was optically centered with the aid of a video camera to insure that no translations were observed as the crystal was rotated through all positions. A unit cell collection was then carried out. After it was determined that the unit cell was not present in the CCDC database a sphere of data was collected. Omega scans were carried out with a 40 sec/frame exposure time and a rotation of 0.50° per frame. The unit cell determination was carried out using the CrystalClear-SM Expert 2.0. Intensity data were corrected for Lorentz, polarization, and background effects using CrysAlisPro[1]. An empirical correction for adsorption was also applied using the program CrysAlisPro[1]. The *SHELXL-2014*[2], series of programs was used for the solution and refinement of the crystal structure. During the initial refinements, an inversion twin was suggested and applied with a resulting BASF value of 0.262. Hydrogen atoms bound to carbon atoms were located in the difference Fourier map where possible and were geometrically constrained using the appropriate AFIX commands. The interstitial dimethyl sulfoxide molecules (3 of the 4 crystallographic unique molecules) were positionally disordered to various degrees within the structure. Of the atom sites that were positionally disordered for DMSO molecules containing S2, S3, and S4, the occupancies of the A and B sites were allowed to free refine to total value of 1. These site occupancies for the A and B positions were 0.86 and 0.14, 0.79 and 0.21, and 0.73 and 0.27, for molecules containing S2, S3, and S4, respectively. To help model this disorder, SIMU, DELU, RIGU (globally) and free variable DFIX distance restraints were applied. The average bond lengths for S-O and S-C in the disordered molecules were 1.442(13) and 1.771(9) Å, respectively.

1. CrysAlisPRO, Oxford Diffraction /Agilent Technologies UK Ltd, Yarnton, England.
2. Sheldrick, G. M. (2015) *Acta Crystallogr.*, C**71**, 3-8.

Table S11. Crystal data and structure refinement for **3**.

Identification code	kre18_01m
Crystal Color	green
Crystal Habit	blade
Empirical formula	C46 H50 B2 N2 O8 S4
Formula weight	908.74
Temperature	100(2) K
Wavelength	0.71073 Å
Crystal system	Monoclinic
Space group	$P2_1$
Unit cell dimensions	a = 8.8155(5) Å alpha = 90 °.
	b = 12.4473(8) Å beta = 92.265(6) °.
	c = 20.7655(13) Å gamma = 90 °.
Volume	2276.8(2) $Å^3$
Z	2
Calculated density	1.326 Mg/m^3
Absorption coefficient	0.264 mm^{-1}
F(000)	956
Crystal size	0.360 x 0.150 x 0.090 mm
Theta range for data collection	1.908 to 25.348 °.
Limiting indices	-10<=h<=10, -14<=k<=14, -24<=l<=24
Reflections collected / unique	17462 / 8293 [R(int) = 0.0644]
Completeness to theta = 25.242°	100.0 %
Refinement method	Full-matrix least-squares on F^2
Data / restraints / parameters	8293 / 696 / 628
Goodness-of-fit on F^2	1.048
Final R indices [I>2sigma(I)]	R1 = 0.0828, wR2 = 0.1732
R indices (all data)	R1 = 0.1412, wR2 = 0.2121
Absolute structure parameter	0.3(2)
Largest diff. peak and hole	0.470 and -0.297 $e.Å^{-3}$

3.11. **2d**×*m*-xylene - Data Collection

Data were collected on a Bruker PLATFORM three circle diffractometer equipped with an APEX II CCD detector and operated at 1350 W (45kV, 30 mA) to generate (graphite monochromated) Mo Kα radiation (λ = 0.71073 Å). Crystals were transferred from the vial and placed on a glass slide in polyisobutylene. A Zeiss Stemi 305 microscope was used to identify a suitable specimen for X-ray diffraction from a representative sample of the material. The crystal and a small amount of the oil were collected on a MīTiGen cryoloop and transferred to the instrument where it was placed under a cold nitrogen stream (Oxford) maintained at 100K throughout the duration of the experiment. The sample was optically centered with the aid of a video camera to ensure that no translations were observed as the crystal was rotated through all positions. A unit cell collection was then carried out. After it was determined that the unit cell was not present in the CCDC database a sphere of data was collected. Omega scans were carried out with a 20 sec/frame exposure time and a rotation of 0.50° per frame. After data collection, the unit cell was re-determined using a subset of the full data collection. Intensity data were corrected for Lorentz, polarization, and background effects using the Bruker program APEX 3. A semi-empirical correction for adsorption was applied using the program *SADABS*[1]. The *SHELXL-2014*[2], series of programs was used for the solution and refinement of the crystal structure. After most of the main atoms sites were identified it was determined that the structure contained both meta-xylene and para-xylene molecules disordered over the same interstitial site. Carbon atoms C43A < C50A and C43B < C50B represent the meta-xylene and para-xylene molecules, respectively. The site occupancies of the two molecules were allowed to refine to a total occupancy of one. The sites occupancies were 0.44 and 0.56 for the meta- and para-molecules, respectively. To help model these overlapping molecules, DFIX, SIMU, and DELU constraints and restraints were applied. Also, due to the exchange of the two xylene molecules, the trifluorobenzyl group containing atoms C27 < C32 and F4 < F6 was positionally disordered and modeled as two sites (A and B) as well. Hydrogen atoms bound to carbon atoms were located in the difference Fourier map and were geometrically constrained using the appropriate AFIX commands. During the initial refinements, the rigid-bond restraint RIGU was applied and was maintained throughout the final refinements.

1. Bruker (2021) SADABS v2016/2. Bruker AXS Inc., Madison, Wisconsin, USA.
2. Sheldrick, G. M. (2015) *Acta Crystallogr*., C**71**, 3-8.

Table S12. Crystal data and structure refinement for **2d**×*m*-xylene.

Identification code	kre18_13
Crystal Color	yellow
Crystal Habit	dendritic
Empirical formula	C50 H34 B2 F6 N2 O4
Formula weight	862.41
Temperature	100(2) K
Wavelength	0.71073 A
Crystal system	Monoclinic
Space group	$P2_1/n$
Unit cell dimensions	a = 13.9353(14) Å alpha = 90 °.
	b = 15.6692(15) Å beta = 107.0980(10) °.
	c = 19.8853(19) Å gamma = 90 °.
Volume	4150.1(7) $Å^3$
Z	4
Calculated density	1.380 Mg/m^3
Absorption coefficient	0.105 mm^{-1}
F(000)	1776
Crystal size	0.480 x 0.460 x 0.060 mm
Theta range for data collection	1.588 to 27.161 °.
Limiting indices	-17<=h<=17, -20<=k<=20, -25<=l<=25
Reflections collected / unique	48069 / 9187 [R(int) = 0.0429]
Completeness to theta = 25.242°	100.0 %
Refinement method	Full-matrix least-squares on F^2
Data / restraints / parameters	9187 / 1072 / 706
Goodness-of-fit on F^2	1.024
Final R indices [I>2sigma(I)]	R1 = 0.0506, wR2 = 0.1277
R indices (all data)	R1 = 0.0789, wR2 = 0.1458
Largest diff. peak and hole	0.695 and -0.391 $e.A^{-3}$

3.12. **2c** - Data Collection

Data were collected on a Bruker PLATFORM three circle diffractometer equipped with an APEX II CCD detector and operated at 1350 W (45kV, 30 mA) to generate (graphite monochromated) Mo Kα radiation (λ = 0.71073 Å). Crystals were transferred from the vial and placed on a glass slide in polyisobutylene. A Zeiss Stemi 305 microscope was used to identify a suitable specimen for X-ray diffraction from a representative sample of the material. The crystal and a small amount of the oil were collected on a MīTiGen cryoloop and transferred to the instrument where it was placed under a cold nitrogen stream (Oxford) maintained at 100K throughout the duration of the experiment. The sample was optically centered with the aid of a video camera to ensure that no translations were observed as the crystal was rotated through all positions.

A unit cell collection was then carried out. After it was determined that the unit cell was not present in the CCDC database a sphere of data was collected. Omega scans were carried out with a 10 sec/frame exposure time and a rotation of 0.50° per frame.

After data collection, the unit cell was re-determined using a subset of the full data collection. Intensity data were corrected for Lorentz, polarization, and background effects using the Bruker program APEX 3. A semi-empirical correction for adsorption was applied using the program *SADABS*[1]. The *SHELXL-2014*[2], series of programs was used for the solution and refinement of the crystal structure. Hydrogen atoms bound to carbon atoms were located in the difference Fourier map and were geometrically constrained using the appropriate AFIX commands. During the final refinement the inconsistent reflection 100 was omitted.

1. Bruker (2021) SADABS v2016/2. Bruker AXS Inc., Madison, Wisconsin, USA.
2. Sheldrick, G. M. (2015) *Acta Crystallogr.*, C**71**, 3-8.

Table S13. Crystal data and structure refinement for **2c**.

Identification code	kre18_20
Crystal Color	yellow
Crystal Habit	blade
Empirical formula	C54 H42 B2 N2 O4
Formula weight	804.51
Temperature	100(2) K
Wavelength	0.71073 A
Crystal system	Monoclinic
Space group	$P2_1/c$
Unit cell dimensions	a = 10.1971(17) Å alpha = 90 °.
	b = 17.232(3) Å beta = 93.292(2) °.
	c = 23.551(4) Å gamma = 90 °.
Volume	4131.4(12) $Å^3$
Z	4
Calculated density	1.293 Mg/m^3
Absorption coefficient	0.080 mm^{-1}
F(000)	1688
Crystal size	0.480 x 0.180 x 0.095 mm
Theta range for data collection	1.465 to 25.452 °.
Limiting indices	-12<=h<=12, -20<=k<=20, -28<=l<=28
Reflections collected / unique	41981 / 7645 [R(int) = 0.0556]
Completeness to theta = 25.242°	100.0 %
Refinement method	Full-matrix least-squares on F^2
Data / restraints / parameters	7645 / 0 / 559
Goodness-of-fit on F^2	1.023
Final R indices [I>2sigma(I)]	R1 = 0.0413, wR2 = 0.0926
R indices (all data)	R1 = 0.0713, wR2 = 0.1062
Largest diff. peak and hole	0.189 and -0.226 $e.A^{-3}$

3.13. **2a** - Data Collection

Data were collected on a Bruker PLATFORM three circle diffractometer equipped with an APEX II CCD detector and operated at 1350 W (45kV, 30 mA) to generate (graphite monochromated) Mo Kα radiation (λ = 0.71073 Å). Crystals were transferred from the vial and placed on a glass slide in polyisobutylene. A Zeiss Stemi 305 microscope was used to identify a suitable specimen for X-ray diffraction from a representative sample of the material. The crystal and a small amount of the oil were collected on a MīTiGen cryoloop and transferred to the instrument where it was placed under a cold nitrogen stream (Oxford) maintained at 100K throughout the duration of the experiment. The sample was optically centered with the aid of a video camera to ensure that no translations were observed as the crystal was rotated through all positions.

A unit cell collection was then carried out. After it was determined that the unit cell was not present in the CCDC database a sphere of data was collected. Omega scans were carried out with a 10 sec/frame exposure time and a rotation of 0.50° per frame.

After data collection, the unit cell was re-determined using a subset of the full data collection. Intensity data were corrected for Lorentz, polarization, and background effects using the Bruker program APEX 3. A semi-empirical correction for adsorption was applied using the program *SADABS*[1]. The *SHELXL-2014*[2], series of programs was used for the solution and refinement of the crystal structure. Hydrogen atoms bound to carbon atoms were located in the difference Fourier map and were geometrically constrained using the appropriate AFIX commands.

1. Bruker (2021) SADABS v2016/2. Bruker AXS Inc., Madison, Wisconsin, USA.
2. Sheldrick, G. M. (2015) *Acta Crystallogr.*, C**71**, 3-8.

Table S14. Crystal data and structure refinement for **2a**.

Identification code	kre18_21
Crystal Color	light orange
Crystal Habit	plate
Empirical formula	C48 H42 B2 N2 O4
Formula weight	573.60
Temperature	100(2) K
Wavelength	0.71073 A
Crystal system	Monoclinic
Space group	$P2_1/n$
Unit cell dimensions	a = 9.8070(13) Å alpha = 90 °.
	b = 14.682(2) Å beta = 103.700(2) °.
	c = 13.0873(17) Å gamma = 90 °.
Volume	1830.7(4) $Å^3$
Z	2
Calculated density	1.329 Mg/m^3
Absorption coefficient	0.083 mm^{-1}
F(000)	772
Crystal size	0.480 x 0.460 x 0.195 mm
Theta range for data collection	2.119 to 27.135 °.
Limiting indices	-12<=h<=12, -18<=k<=18, -16<=l<=16
Reflections collected / unique	21091 / 4044 [R(int) = 0.0259]
Completeness to theta = 25.242°	100.0 %
Refinement method	Full-matrix least-squares on F^2
Data / restraints / parameters	4044 / 0 / 256
Goodness-of-fit on F^2	1.055
Final R indices [I>2sigma(I)]	R1 = 0.0365, wR2 = 0.0879
R indices (all data)	R1 = 0.0445, wR2 = 0.0930
Largest diff. peak and hole	0.381 and -0.209 $e.A^{-3}$

3.14. **2d**×mesitylene - Data Collection

Data were collected on a Bruker PLATFORM three circle diffractometer equipped with an APEX II CCD detector and operated at 1350 W (40kV, 30 mA) to generate (graphite monochromated) Mo Kα radiation (λ = 0.71073 Å). Crystals were transferred from the vial and placed on a glass slide in polyisobutylene. A Zeiss Stemi 305 microscope was used to identify a suitable specimen for X-ray diffraction from a representative sample of the material. The crystal and a small amount of the oil were collected on a MīTiGen cryoloop and transferred to the instrument where it was placed under a cold nitrogen stream (Oxford) maintained at 100K throughout the duration of the experiment. The sample was optically centered with the aid of a video camera to ensure that no translations were observed as the crystal was rotated through all positions.

A unit cell collection was then carried out. After it was determined that the unit cell was not present in the CCDC database a sphere of data was collected. Omega scans were carried out with a 60 sec/frame exposure time and a rotation of 0.50° per frame.

After data collection, the unit cell was re-determined using a subset of the full data collection. Intensity data were corrected for Lorentz, polarization, and background effects using the Bruker program APEX 3. A semi-empirical correction for adsorption was applied using the program *SADABS*[1]. The *SHELXL-2014*[2], series of programs was used for the solution and refinement of the crystal structure. Hydrogen atoms bound to carbon atoms were located in the difference Fourier map and were geometrically constrained using the appropriate AFIX commands. During the final refinements, the inconsistent reflection 3-24 was omitted and an extinction coefficient was applied. Also with the structure, there were three crystallographically unique molecules, resulting in a Z' value of 3 for the structure.

1. Bruker (2021) SADABS v2016/2. Bruker AXS Inc., Madison, Wisconsin, USA.
2. Sheldrick, G. M. (2015) *Acta Crystallogr.*, C**71**, 3-8.

Table S15. Crystal data and structure refinement for **2d**×mesitylene.

Identification code	kre18_28
Crystal Color	yellow
Crystal Habit	blade
Empirical formula	C198 H168 B6 F18 N6 O12
Formula weight	3230.23
Temperature	100(2) K
Wavelength	0.71073 A
Crystal system	Triclinic
Space group	$P\bar{1}$
Unit cell dimensions	a = 13.422(3) Å alpha = 112.644(3) °.
	b = 18.598(4) Å beta = 101.792(3) °.
	c = 18.845(4) Å gamma = 101.202(3) °.
Volume	4051.4(15) Å^3
Z	1
Calculated density	1.324 Mg/m^3
Absorption coefficient	0.095 mm^{-1}
F(000)	1686
Crystal size	0.480 x 0.185 x 0.090 mm
Theta range for data collection	1.231 to 25.429 °.
Limiting indices	-16<=h<=16, -22<=k<=22, -22<=l<=22
Reflections collected / unique	48631 / 14887 [R(int) = 0.0760]
Completeness to theta = 25.242°	99.9 %
Refinement method	Full-matrix least-squares on F^2
Data / restraints / parameters	14887 / 0 / 1094
Goodness-of-fit on F^2	1.016
Final R indices [I>2sigma(I)]	R1 = 0.0669, wR2 = 0.1592
R indices (all data)	R1 = 0.1286, wR2 = 0.1869
Extinction coefficient	0.0059(5)
Largest diff. peak and hole	0.302 and -0.288 e.A^{-3}

3.15. **2d**×*o*-xylene - Data Collection

Data were collected on a Bruker PLATFORM three circle diffractometer equipped with an APEX II CCD detector and operated at 1350 W (40kV, 30 mA) to generate (graphite monochromated) Mo Kα radiation (λ = 0.71073 Å). Crystals were transferred from the vial and placed on a glass slide in polyisobutylene. A Zeiss Stemi 305 microscope was used to identify a suitable specimen for X-ray diffraction from a representative sample of the material. The crystal and a small amount of the oil were collected on a MīTiGen cryoloop and transferred to the instrument where it was placed under a cold nitrogen stream (Oxford) maintained at 100K throughout the duration of the experiment. The sample was optically centered with the aid of a video camera to ensure that no translations were observed as the crystal was rotated through all positions.

A unit cell collection was then carried out. After it was determined that the unit cell was not present in the CCDC database a sphere of data was collected. Omega scans were carried out with a 30 sec/frame exposure time and a rotation of 0.50° per frame.

After data collection, the unit cell was re-determined using a subset of the full data collection. Intensity data were corrected for Lorentz, polarization, and background effects using the Bruker program APEX 3. A semi-empirical correction for adsorption was applied using the program *SADABS*[1]. The *SHELXL-2014*[2], series of programs was used for the solution and refinement of the crystal structure. Hydrogen atoms bound to carbon atoms were located in the difference Fourier map and were geometrically constrained using the appropriate AFIX commands.

1. Bruker (2021) SADABS v2016/2. Bruker AXS Inc., Madison, Wisconsin, USA.
2. Sheldrick, G. M. (2015) *Acta Crystallogr.*, C**71**, 3-8.

Table S16. Crystal data and structure refinement for **2d**×*o*-xylene.

Identification code	kre18_36
Crystal Color	yellow
Crystal Habit	blocky
Empirical formula	C58 H44 B2 F6 N2 O4
Formula weight	968.57
Temperature	100(2) K
Wavelength	0.71073 A
Crystal system	Triclinic
Space group	$P\bar{1}$
Unit cell dimensions	a = 12.725(2) Å alpha = 70.841(5) °.
	b = 13.930(2) Å beta = 69.921(5) °.
	c = 16.092(3) Å gamma = 65.228(4) °.
Volume	2375.1(7) $Å^3$
Z	2
Calculated density	1.354 Mg/m^3
Absorption coefficient	0.100 mm^{-1}
F(000)	1004
Crystal size	0.480 x 0.350 x 0.240 mm
Theta range for data collection	1.380 to 27.146 °.
Limiting indices	-16<=h<=16, -17<=k<=17, -20<=l<=20
Reflections collected / unique	54601 / 10511 [R(int) = 0.0287]
Completeness to theta = 25.242°	100.0 %
Refinement method	Full-matrix least-squares on F^2
Data / restraints / parameters	10511 / 0 / 653
Goodness-of-fit on F^2	1.023
Final R indices [I>2sigma(I)]	R1 = 0.0371, wR2 = 0.0855
R indices (all data)	R1 = 0.0496, wR2 = 0.0925
Largest diff. peak and hole	0.370 and -0.240 $e.A^{-3}$

3.16. **4** - Data Collection

Data were collected on a Rigaku XtaLAB Synergy-*i* Kappa diffractometer equipped with a PhotonJet-*i* X-ray source operated at 50 W (50kV, 1 mA) to generate Cu Kα radiation (λ = 1.54178 Å) and a HyPix-6000HE HPC detector. Crystals were transferred from the vial and placed on a glass slide in type NVH immersion oil by Cargille. A Zeiss Stemi 305 microscope was used to identify a suitable specimen for X-ray diffraction from a representative sample of the material. The crystal and a small amount of the oil were collected on a Hampton Research 20-micron nylon CryoLoop and transferred to the instrument where it was placed under a cold nitrogen stream (Oxford 700 series) maintained at 100K throughout the duration of the experiment. The sample was optically centered with the aid of a video camera to ensure that no translations were observed as the crystal was rotated through all positions. A unit cell collection was then carried out. After it was determined that the unit cell was not present in the CCDC database a data collection strategy was calculated by *CrysAlis^Pro*[1]. After data collection, the unit cell was re-determined using a subset of the full data collection. Intensity data were corrected for Lorentz, polarization, and background effects using the *CrysAlis^Pro*[1]. A numerical absorption correction was applied based on a Gaussian integration over a multifaceted crystal and followed by a semi-empirical correction for adsorption applied using the program *SCALE3 ABSPACK*[2]. The *SHELX-2018*[3], series of programs was used for the solution and refinement of the crystal structure within OLEX2 software[4]. While the molecule of interest was fairly defined, there was a considerable amount of space with the structure that was occupied by ordered and disordered acetone and water molecules. Due to the positional disorder of the interstitial acetone (O7, C43 < C45) and water (O8) molecules in the structure, SIMU, RIGU, and free variable restraints were applied where necessary. The hydrogen atom positions on the water molecule were not located or added to the structure. Hydrogen atoms bound to carbon atoms were geometrically constrained using the appropriate AFIX commands.

1. CrysAlis^Pro (2018) Oxford Diffraction Ltd.
2. SCALE3 ABSPACK (2005) Oxford Diffraction Ltd.
3. Sheldrick, G. M. (2015) *Acta Crystallogr*., C**71**, 3-8.
4. Dolomanov, O. V.; Bourhis, L. J.; Gildea, R. J.; Howard, J. A. K.; Puschmann. H. (2009) *J. Appl. Cryst*. **42**, 339-341.

Table S17. Crystal data and structure refinement for **4**.

**Compound**	**kre21_08**
Formula	$C_{85}H_{74}B_4N_4O_{12.67}$
$D_{calc.}$/ g cm^{-3}	1.281
μ/mm^{-1}	0.684
Formula Weight	1397.38
Colour	clear yellow
Shape	irregular-shaped
Size/mm^3	0.13×0.10×0.09
T/K	99.9(5)
Crystal System	trigonal
Space Group	*R*-3
a/Å	32.0075(2)
b/Å	32.0075(2)
c/Å	18.3787(2)
α/°	90
β/°	90
γ/°	120
V/Å^3	16306.0(3)
Z	9
Z'	0.5
Wavelength/Å	1.54184
Radiation type	Cu K_α
Θ_{min}/°	2.761
Θ_{max}/°	77.321
Measured Refl's.	34158
Indep't Refl's	7356
Refl's I≥2 σ(I)	6530
R_{int}	0.0237
Parameters	516
Restraints	42
Largest Peak	0.529
Deepest Hole	-0.440
GooF	1.046
wR_2 (all data)	0.1609
wR_2	0.1571
R_1 (all data)	0.0597
R_1	0.0544
